# Maintaining Grain Boundary Segregation-Induced Strengthening Effect in Extremely Fine Nanograined Metals

**DOI:** 10.1021/acs.nanolett.5c01032

**Published:** 2025-03-24

**Authors:** Lei Qian, Jiacheng Zhang, Wenqing Yang, Yunjiang Wang, Kangcheung Chan, Xu-Sheng Yang

**Affiliations:** † Department of Industrial and Systems Engineering, Research Institute for Advanced Manufacturing, The Hong Kong Polytechnic University, Hung Hom, Kowloon, Hong Kong 999077, China; ‡ Hong Kong Polytechnic University Shenzhen Research Institute, Shenzhen 518060, China; § State Key Laboratory of Nonlinear Mechanics, Institute of Mechanics, Chinese Academy of Sciences, Beijing 100080, China

**Keywords:** Grain boundary segregation, amorphous grain boundary, extremely fine grain size, deformation mechanism, strengthening effect

## Abstract

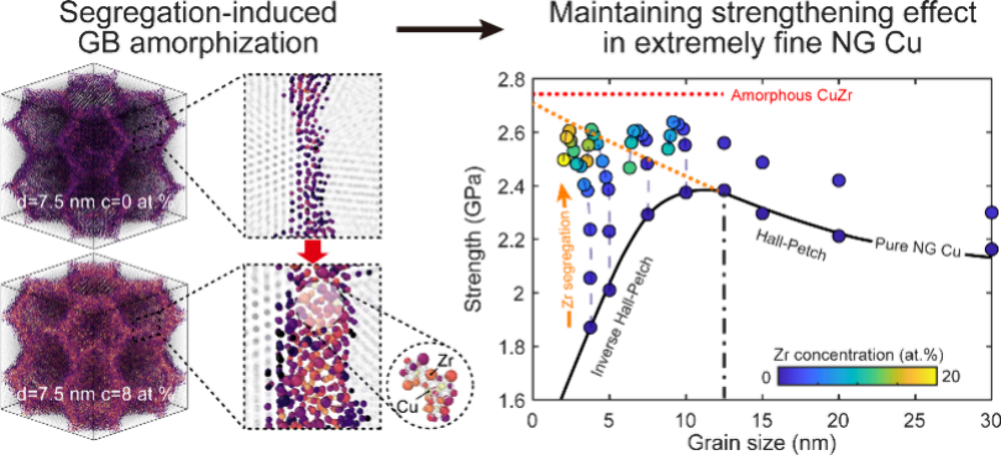

Reinforcing grain boundaries through solute segregation is a promising strategy to strengthen nanograined metals. However, maintaining strengthening in extremely fine nanograined metals poses challenges due to grain size reduction and grain boundary structural changes from excessive segregation. This study employs hybrid Monte Carlo/Molecular Dynamics simulations to investigate the interplay between solute concentration, grain boundary structure, deformation mechanism, and strength in Zr-segregated nanograined Cu. Results exhibit significant strength enhancement by optimizing segregation, extending the strengthening effect to a grain size as small as 3.75 nm. Continuous Zr segregation induces a progressive transition from original grain boundaries to segregated and ultimately amorphous grain boundaries. Amorphization alters the dominant deformation mechanism from grain boundary migration to homogeneous shear-transformation-zone activation, fostering and optimizing the strengthening effect in extremely fine nanograined Cu. These findings inspire a novel approach of segregation-induced grain boundary amorphization to leverage strong boundaries and extremely fine nanograins for strengthening nanograined metals.

Nanograined (NG) metals, characterized by nanoscale grain sizes, exhibit exceptional strength due to high-density grain boundaries (GBs).^1,2^ These GBs serve as active sites for various atomic-scale deformation processes, including GB diffusion, migration, sliding, and dislocation nucleation/pileup at GB, thereby affecting mechanical behaviors.^3−5^ Refining nanograins is a key strategy to leverage GB strengthening following the classical Hall-Petch relationship.^6−8^ However, the inherent instability of GBs and associated GB-mediated mechanisms intensify when reducing the grain size into the extremely fine regime (typically below 10 nm), causing detrimental softening rather than strengthening.^9,10^

Various strategies such as alloying,^11^ adopting low-energy boundaries,^12−14^ and mechanically/thermally induced GB relaxation,^15,16^ have been employed to maintain the Hall-Petch strengthening at smaller grain sizes. Particularly, GB segregation, featured by preferential accumulation of solute elements at GBs, has shown promise for reinforcing NG metals.^17−19^ Hu et al. demonstrated that NG Ni with Mo-segregated GBs, prepared via annealing, achieved exceptional hardness and extended the Hall-Petch relationship to grain sizes below 10 nm.^10^ Mo segregation stabilizes GBs by suppressing GB-mediated plastic processes, which become more pronounced at smaller grain sizes with higher Mo concentrations. These studies highlight GB segregation’s potential to maintain strengthening in NG metals, even at extremely fine grain sizes with significantly amplified GB volume fractions. In the pursuit of maximizing the benefits of GB segregation, higher solute concentrations are often introduced. However, excessive segregation can disrupt the original GB structure, partially or completely transforming it into crystalline second phases, forming dual-phase nanostructures,^11,20^ or into amorphous phases, resulting in crystalline–amorphous nanocomposites.^21,22^ Such “phase-like” GB transition can either strengthen or weaken materials, depending on resultant nanostructures and defects.^11,23,24^ Nanosized amorphous phases, containing few defects, have proven more effective than their crystalline counterparts in improving strength, plasticity, and thermal stability, as demonstrated in a series of studies using amorphous ceramics SiOC as GBs.^25−28^ Meanwhile, amorphous metals, characterized by structural heterogeneities at the nanoscale, offer critical insights into their structure–property relationships.^29,30^ This concept of structural heterogeneity has been extended to amorphous GBs in NG metals by evaluating short-range orders (SROs), emphasizing the impact of local GB structure on mechanical properties.^31,32^

Segregation-induced GB amorphization has been observed in various binary, ternary, and multicomponent nanostructured alloys,^33−37^ revealing opportunities to further explore its strengthening potential. Li et al. progressively strengthened Al–Cr alloys through Cr segregation-induced multistage phase transformations, achieving peak strength at the transition to a crystalline–amorphous nanostructure with grain sizes within the Hall-Petch strengthening regime.^21^ However, the role of segregation-induced amorphous GBs in the mechanical response of extremely fine NG metals, where deformation is predominantly governed by GB-mediated mechanisms, remains unexplored. A comprehensive investigation on the interplay between solute concentration, GB structure (particularly in terms of SROs), and strengthening effect is necessary. Meanwhile, deeper insights into deformation mechanisms of extremely fine NG metals with amorphous GBs are crucial to fully harness their strengthening potential. Note that experimental manipulation and characterization of GB segregation/amorphization remain challenging, especially in extremely fine NG metals,^22,38−40^ limiting insights into intrinsic GB structure and underlying mechanistic rationale. Alternatively, this study employs hybrid Monte Carlo/Molecular Dynamics (MC/MD) simulations to explore the influence of Zr segregation on GB structures and strength performance of NG Cu. By varying solute concentrations, we achieve sequential transitions from original to segregated GBs and ultimately to amorphous GBs, elucidating distinct deformation mechanisms and strength performance. Notably, the optimal strength occurs when GBs transform into fully amorphous state, with maximum strength enhancement shown to be grain size-dependent.

High-density GBs critically govern deformation behavior and strength enhancement, highlighting the importance of GB utilization for strengthening.^41^ Here, via hybrid MC/MD simulations, intentional GB segregation of Zr solute was applied to engineer GBs in pure NG Cu for strengthening (Figure 1a). Details on material selection, atomic configuration, tensile tests, and visualization methods are provided in Supporting Information, Texts S1–S3. Given observed GB softening below 12.5 nm (see Supporting Information, Text S4), we focused on grain size regime of 3.75–10 nm to study Zr segregation effects. Initial homogeneous Zr distribution within interfacial regions (Figure 1a), combined with the abundance of high-angle GBs, facilitates uniform Zr segregation along GB networks (see Supporting Information, Text S5). These networks exhibit Zr concentration-dependent structural and compositional transitions, as shown in Figure 1b. Figure 1c displays the stress–strain curves of Zr-segregated extremely fine NG Cu, revealing the substantial enhancement in flow stress. As further shown in Figure 1d, Zr-segregated counterparts demonstrate significant strength enhancement across all tested grain sizes and Zr concentrations. Notably, GB segregation achieves predicated Hall-Petch strength (orange dashed line) even in softening regimes, approaching the strength of the amorphous Cu_80_Zr_20_ nanostructure (see Supporting Information, Text S6). While excessive segregation induces GB thickening and grain size reduction (discussed later), the maximum strength enhancement increases with decreasing initial grain size, demonstrating an improved capacity to utilize high-density GBs for strengthening.

**Figure 1:**
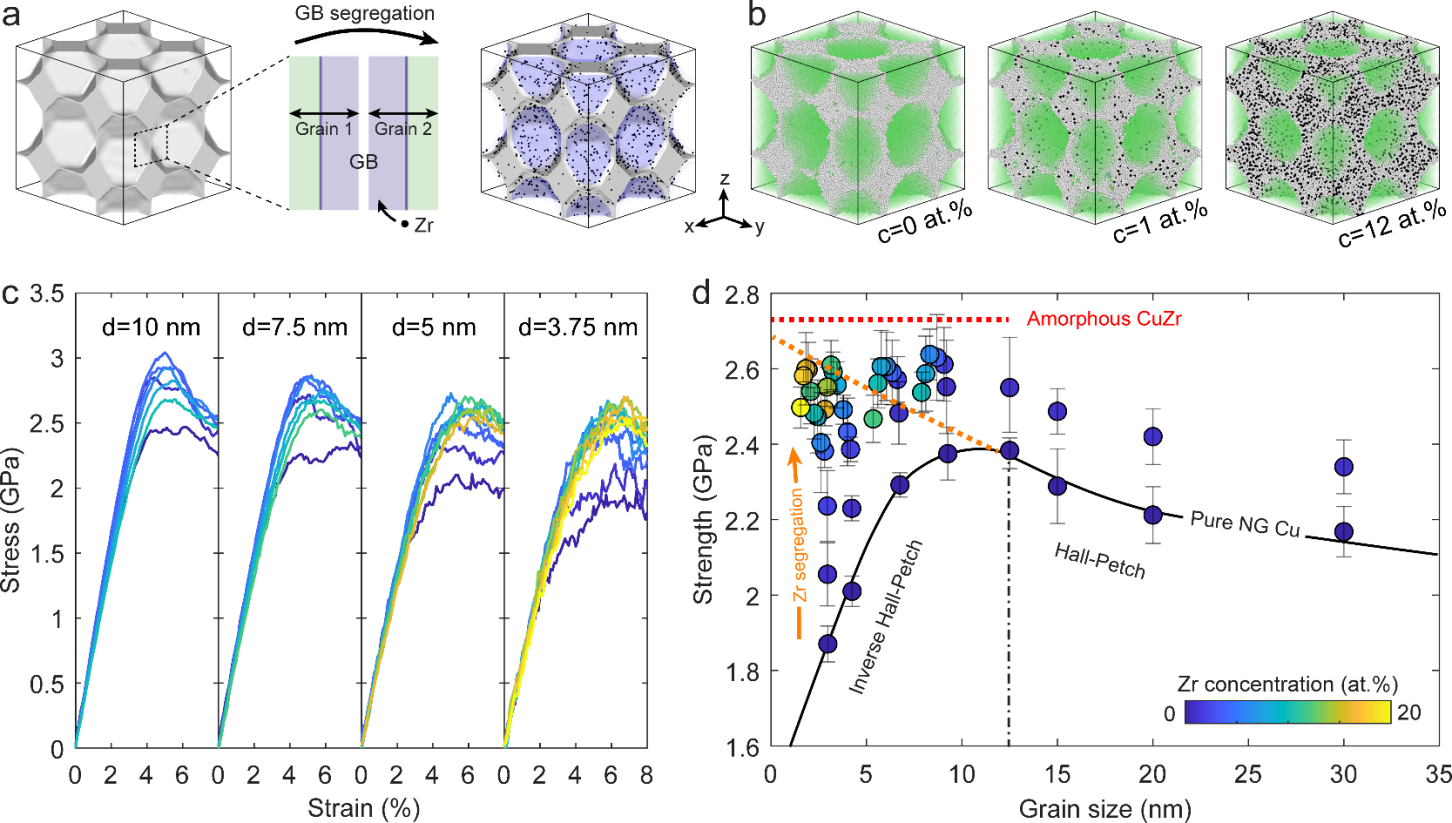
GB segregation-induced strengthening in extremely fine NG Cu. (a) Schematic illustration of intentional Zr segregation at interfacial regions to promote uniform Zr distribution and GB transition. The original GB network is reconstructed with gray color. Blue interfaces indicate boundaries of segregation region while segregated Zr atoms are shown in black. (b) Homogeneous GB networks formed at varying total Zr concentrations, with Zr atoms highlighted in black and lattice atoms shown semitransparent for clarity. (c) Stress–strain curves of Zr-segregated NG Cu with varying grain sizes and total Zr concentration. (d) Variations of strength as functions of grain size and Zr concentrations for pure NG Cu and segregated NG Cu. The orange dashed line extrapolates the Hall–Petch relation (simulated strength vs grain size) into softening regime, representing predicted Hall–Petch strengths for extremely fine NG Cu. The red dashed line represents the strength of amorphous Cu_80_Zr_20_ nanostructure prepared at cooling rates of 10^9^ K s^–1^.

Experimental studies using transmission electron microscopy (TEM) and atom probe tomography (APT) have demonstrated diverse behaviors of Zr doping in Cu, including homogeneous segregation along GBs,^42^ cluster formation,^39^ and amorphization,^43^ depending on processing routes and compositions.^22^ Akin to experimental analyses, we examine individual GBs in representative bicrystal regions at varying total Zr concentrations (Figure 2a). At low Zr content (≤2 at. % for a initial grain size of 7.5 nm), Zr atoms distribute discretely and homogeneously along the original GBs, indicating well-defined segregation. When the total Zr concentration exceeds ∼2 at. %, GB thickness starts to increase, indicating the saturation of segregated GBs and GB transition onset. At higher Zr concentrations (≥4 at. % for a grain size of 7.5 nm), GB transition becomes pronounced, resulting in significant GB thickening and disorder. Variations in local Zr concentration across representative bicrystal regions (Figure S5) show a sharp increase in Zr enrichment and GB thickness at concentrations above 2 at. %, indicating the initiation of GB amorphization.

**Figure 2:**
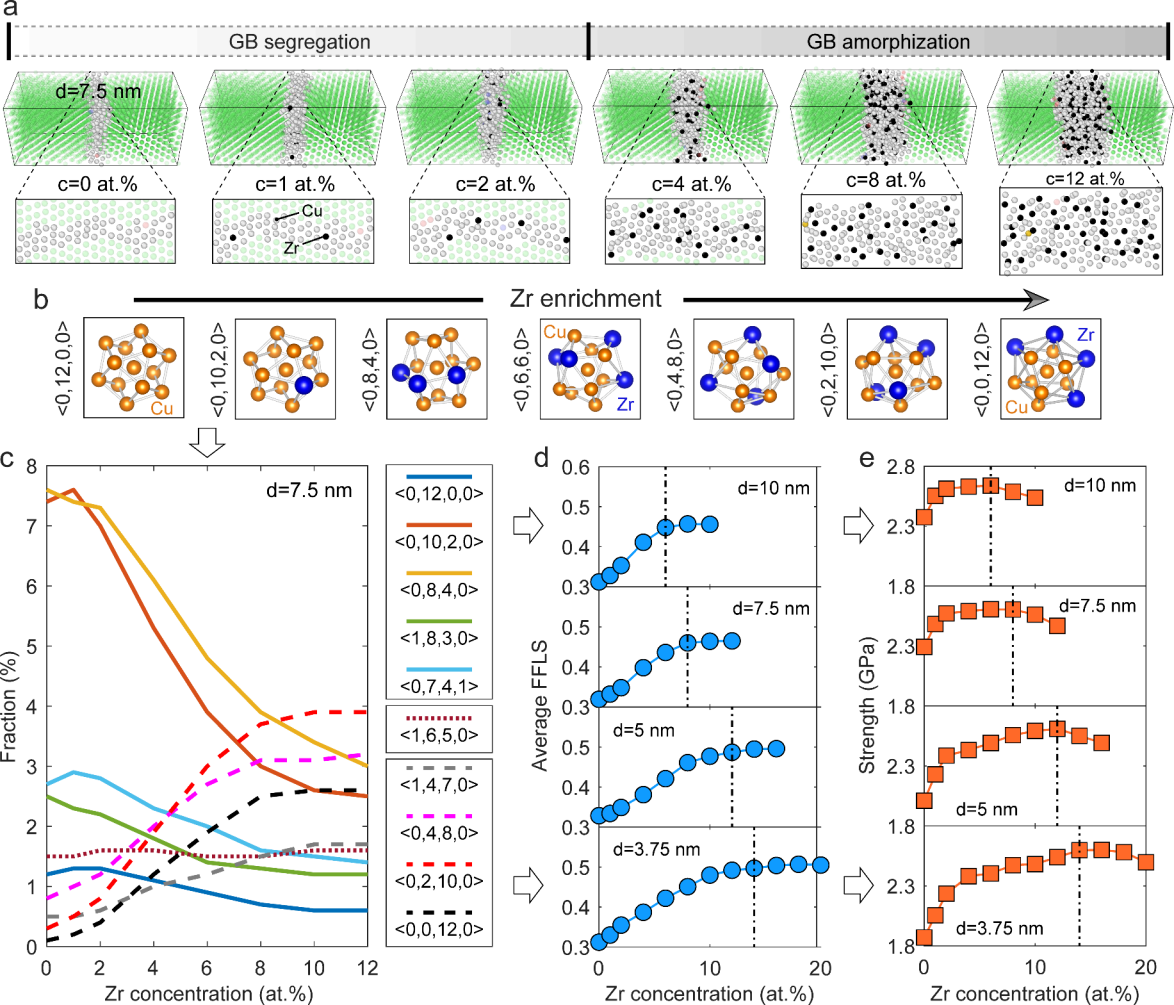
Continuous Zr-segregation-induced GB transitions in NG Cu with grain size of 7.5 nm. (a) Representative bicrystal regions illustrating the GB transition from GB segregation to GB amorphization with increasing total Zr concentrations. (b) Schematic illustrations of representative SROs with increasing Zr enrichment. (c) Evolution of fractions of representative SROs with increasing Zr concentration. (d) Evolutions of average FFLS with increasing total Zr concentration. (e) Evolutions of strength with increasing total Zr concentration. The dotted-dashed lines in (d, e) indicate the critical Zr concentration required to form fully amorphous GBs.

To quantitatively characterize Zr enrichiment-induced GB structural/compositional transitions, we analyze average GB thickness and composition across GB networks as functions of total Zr concentration (Figure S6). Additionally, the McLean model was employed to predict theoretical local Zr concentrations at GBs and define the critical Zr concentration for onset of GB amorphization via comparing with simulated results (see Supporting Information, Text S7). The analysis of overall GB networks consistently identifies a critical total Zr concentration of ∼2.5 at. % for inducing GB amorphization at an initial grain size of 7.5 nm, within the 2 to 4 at. % range observed from individual GB analysis in Figure 2a. This critical concentration for GB amorphization reflects the thermodynamic-kinetic balance where Zr segregation lowers local GB energy sufficiently to overcome the energy penalty of amorphization,^17,35^ while rapid cooling kinetically stabilizes disordered configurations by suppressing crystallization.^22^ These findings align with experimental observations in Zr-doped NG Cu, where TEM and APT analyses reveal distinct stages of GB transition—from well-defined segregation to amorphous cluster and finally to thick amorphous network with increasing Zr concentration.^39^ Excessive Zr enrichment elevates local Zr concentrations, driving progressive amorphization and broadening of amorphous-dominated GBs. Simulation results provide atomistic insights into the same trends and mechanisms observed experimentally, revealing the transition from GB segregation to amorphization due to local Zr enrichment.

To discern intrinsic differences in GB structures at varying Zr concentrations, we evaluate their local structural units of SROs identified through the Voronoi tessellation method (see Supporting Information, Text S8).^32^ Considering the individual GB as a repetition and connection of different local structures, dominant SROs with high fractions describe the preferred atomic arrangement of formed GB, which vary with the evolution of Zr enrichment, as illustrated in Figure 2b. According to calculated fractions of atoms associated with the ten most frequent SROs (see Supporting Information, Text S8 and Figure S7), the evolution of representative SRO fractions as a function of Zr concentration is presented in Figure 2c. Across the studied concentration range, a general trend can be observed: the predominance of crystalline-like SROs (i.e., ⟨0, 12, 0, 0⟩, ⟨0, 10, 2, 0⟩, and ⟨0, 8, 4, 0⟩) at low concentration is gradually replaced by amorphous-like SROs (i.e., ⟨0, 0, 12, 0⟩, ⟨0, 2, 10, 0⟩, and ⟨0, 4, 8, 0⟩) at higher concentrations. A similar trend is observed for other extremely fine grain sizes, as shown in Figure S8. SRO analyses reveal that local atomic arrangements of highly segregated GBs resemble those of amorphous phases, suggesting the formation of amorphous-dominated GBs. The population of the amorphous-like SROs in a given system can be quantified using the average five-fold local symmetry (FFLS) (see Supporting Information, Text S8), which serves as an indicator of amorphous state.^44^ As shown in Figure 2d, FFLS values for all grain sizes exhibit concentration-dependent behavior characterized by rapid increases followed by saturation at constant values, confirming the progressive GB amorphization driven by Zr enrichment and attainment of a fully amorphous state at high Zr concentrations. Accordingly, the dependence of flow strength on Zr concentration, depicted in Figure 2e, shows a gradual rise toward a maximum at a critical Zr concentration, followed by a slight decrease at higher concentrations. By correlating the calculated SRO fraction and average FFLS, it becomes evident that maximum strength occurs when amorphous-like SROs dominate, demonstrating segregation-induced GB amorphization optimizes strengthening at critical concentrations for achieving fully amorphous GBs, which obviously vary with grain sizes.

As the predominant deformation carrier in extremely fine NG Cu, the specific GB structure determines its overall deformation mechanism. One crucial role of GBs is serving as highly effective sources and sinks for dislocations, interacting with GB during deformation.^45−47^ Upon reducing grain size into the softening regime (here, below 12.5 nm), dislocation activity in pure NG Cu becomes predominantly localized at or near GBs rather than in grain interiors (GIs), as evidenced by dislocation line distribution in Figure S9. When segregating Zr into extremely fine NG Cu and increasing total Zr concentrations, both pre-existing dislocations in undeformed samples and newly formed dislocations in deformed samples decrease across all studied grain sizes, confirmed by corresponding statistics on dislocation line length in Figure S10. Detailed discussions on grain size/Zr concentration-dependent dislocation-GB interaction are provided in Supporting Information, Text S9. These segregation-induced variations in dislocation-GB interaction may yield a transition of predominant mechanism in extremely fine NG Cu.

Figure 3 compares dislocation and shear strain distributions at different total Zr concentrations for NG Cu with a grain size of 7.5 nm. The undeformed pure NG sample (Figure 3a1) exhibits numerous pre-existing dislocations at or near GBs, which act as primary sites for dislocation nucleation. A magnified view of a representative GB during deformation (Figure 3b1) shows significant structural fluctuations, indicating GB softening mechanisms like GB sliding or migration driven by dislocation-GB interactions. Upon segregating Zr at GBs (2 at. %), the number of pre-existing dislocations is reduced (Figure 3a2), resulting in a more stable GB, as seen in the comparison of magnified GBs in Figure 3b1 and Figure 3b2. Theoretical studies suggest that solute atoms segregated at the GBs exert a pinning effect, making dislocation nucleation more difficult.^48^ However, in GBs with low Zr concentrations, iterative dislocation processes can still induce GB structural fluctuations and instability, as shown by black circles in Figure 3c1,c2 and high strain concentrations in Figure 3d1,d2. In contrast, with sufficient Zr segregation (8 at. %) to form amorphous GBs, dislocation-related processes are significantly suppressed. As shown in Figure 3a3, the sample with amorphous GBs contains fewer pre-existing dislocations than segregated counterparts, consistent with previous simulation studies on GB disorder effects.^49^ Additionally, it is reported that the formation of amorphous intergranular films can reduce GB energy to enhance GB stability, thus decreasing the driving force for GB migration.^22^ As reflected in even shear strain distribution (Figure 3d3), amorphous GBs deform uniformly without noticeable structural distortion. These findings suggest that GB softening mechanisms, typically driven by dislocation-GB interactions, are significantly alleviated in samples with amorphous GBs. Accordingly, a steady decrease in average GB shear strain magnitude is observed, reaching saturation upon amorphous GB formation at critical Zr concentrations (Figure S11). This homogeneous deformation, characterized by few dislocation-GB interactions and even strain distribution, dominates the deformation behavior of extremely fine NG Cu with amorphous GBs.

**Figure 3:**
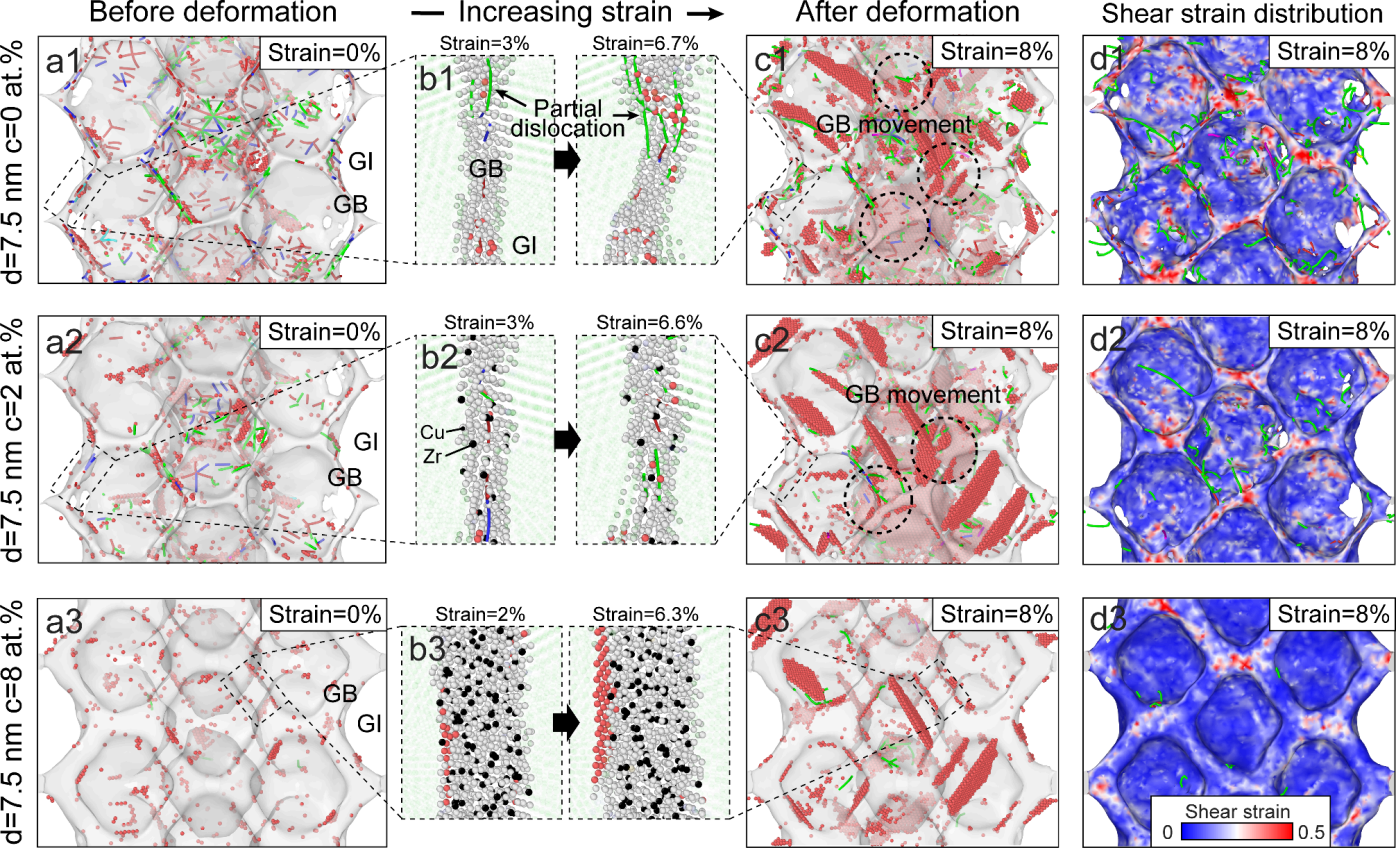
Segregation-induced transition of deformation mechanisms in NG Cu with a grain size of 7.5 nm. (a) Cross-sectional snapshots illustrating dislocation distribution before deformation (at 0% strain). (b) Magnifications of representative GBs displaying structural evolution during deformation. Segregated Zr atoms at GBs are visualized with black color. (c) Cross-sectional snapshots illustrating dislocation distribution after deformation (at 8% strain). Black circles indicate dislocation-induced structural fluctuations of unstable GBs. In (a–c), only GB atoms or reconstructed GB networks in white color and hcp atoms in red color are displayed for clarity. (d) Shear strain distribution of GB regions after deformation (at 8% strain). Green lines indicate Shockley partial dislocations.

Depending on local structures, GBs exhibit varying susceptibility to atomistic processes during deformation.^22,23^Figure 4a shows the spatial distribution of calculated FFLS within GB networks before deformation, approximating SRO distribution across Zr concentrations. Brighter atoms with FFLS values close to 1 represent amorphous-like SROs, which increasingly populate GB sites with higher Zr concentrations, as shown in magnified GB views (Figure 4b). Specifically, the amorphous GB in Figure 4b3 displays a gradient SRO distribution, transitioning from crystalline-like SROs near interfaces to amorphous-like SROs in interiors. To evaluate local irreversible rearrangement in deformed GBs,^8^Figure 4c shows the distribution of nonaffine squared displacements *D*^2^ of GB atoms after deformation. In original (Figure 4c1) and segregated GBs (Figure 4c2), relatively high *D*^2^ are observed across GBs, indicative of widespread atomic rearrangement and low resistance to deformation. In contrast, amorphous GBs (Figure 4c3) demonstrate markedly lower *D*^2^ in highly Zr-segregated regions containing amorphous-like SROs, e.g., ⟨0,0,12,0⟩. The correlation between *D*^2^ and FFLS demonstrates that amorphous GB interior atoms with higher FFLS offer enhanced deformation resistance. Simulation studies have shown that in amorphous structures, regions with lower FFLS accommodate larger plastic deformation, while those with higher FFLS resist atomic rearrangements.^50^ The nanosized thickness and spatial distribution of SROs within amorphous GBs introduce local structural heterogeneity,^29,51^ providing alternative pathways for atomistic processes to accommodate deformation. Figure 4d illustrates atomic movements at representative GBs during deformation. In original GBs without Zr segregation (Figure 4d1), obvious atomic displacements are primarily associated with GB migration driven by dislocation-GB interactions (black arrows), similar to simulations on pure NG Ni.^52^ Repeated dislocation nucleation and annihilation at many GB sites can facilitate GB migration, shifting them significantly from their original position, as evidenced by increased distance between blue GB plane and black reference plane. Figure 4d2 shows that Zr-segregated GBs exhibit alleviated migration, manifesting as partial GB migration after deformation. Only specific GB sites lacking Zr segregation and exhibiting low resistance are susceptible to migration upon dislocation absorption or nucleation at adjacent sites, highlighting GB segregation’s role in suppressing dislocation-GB interactions and reducing GB mobility during deformation.^10^

**Figure 4:**
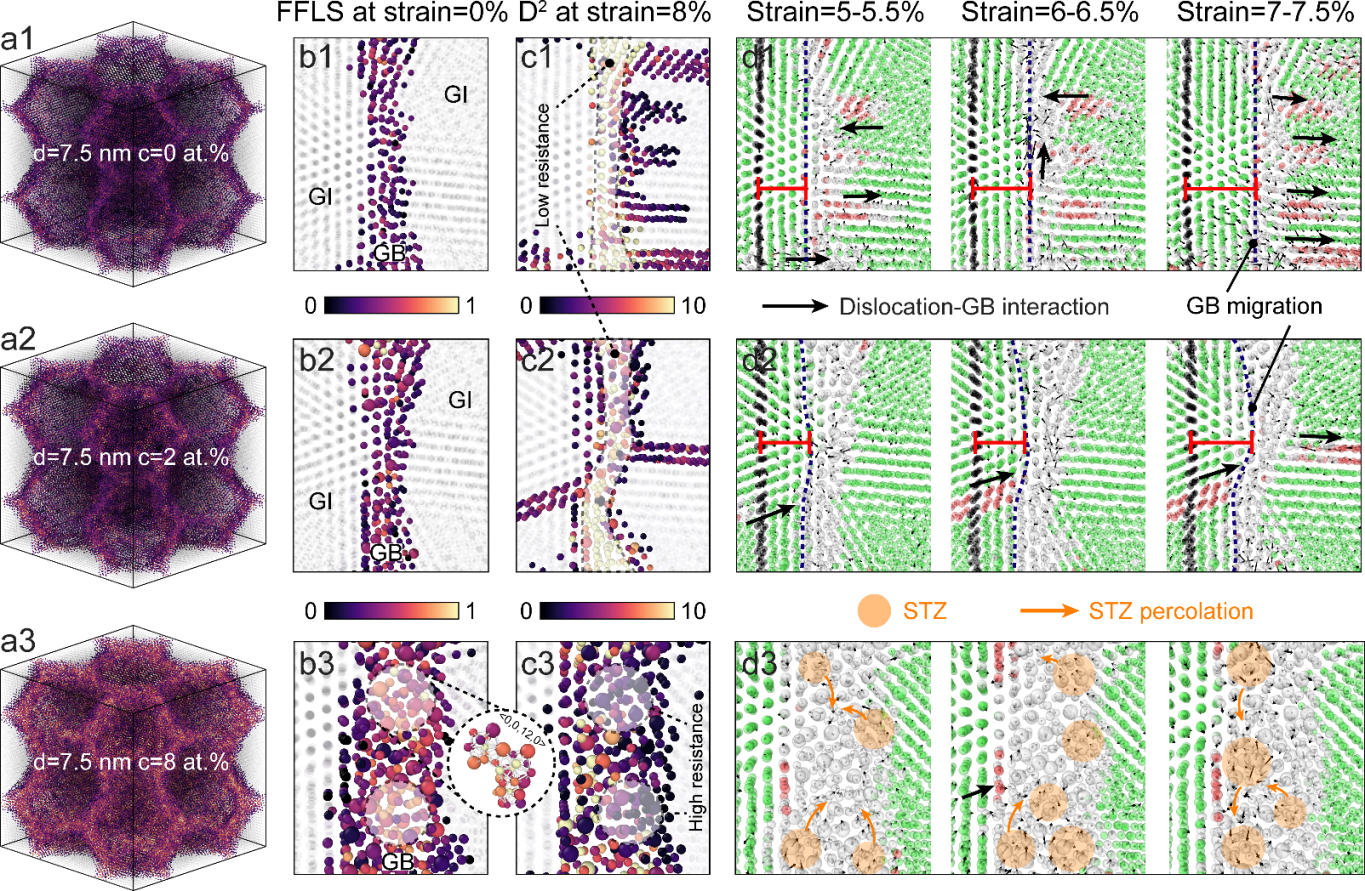
Atomistic mechanisms of various GBs during deformation in samples with a grain size of 7.5 nm. (a) MD snapshots of FFLS distribution in GB networks before deformation. (b) Cross-sectional snapshots of FFLS distribution in representative GBs before deformation. In (a) and (b), atoms are colored according to calculated FFLS. Atoms with maximum value of 1 represent SROs of ⟨0,0,12,0⟩, as illustrated in the magnification. (c) Cross-sectional snapshots of *D*^2^ distribution in representative GBs after deformation. (d) Atomic displacements of GB atoms during deformation. In d1 and d2, the black reference plane is used to indicate GB migration via estimating its distance relative to the deformed GB.

Homogeneous deformation can be achieved through GB amorphization induced by substantial Zr segregation, which significantly reduces dislocation-GB interactions and shifts the deformation primarily to occur within GBs. The realization of plastic flow in nanometer-sized amorphous structures relies on shear-transformation-zone (STZ) activation and percolation.^53−55^ As shown in Figure 4d3, local atomic movements within amorphous GBs initiate in low-resistance interfacial regions and propagate toward higher-resistance areas. Residual GB dislocations are either blocked or absorbed by amorphous GBs, inducing localized strain that can be easily diffused by interfacial STZ activations, consistent with experimentally observed plastic flow in CuZr nanostructures with amorphous intergranular films.^22^ Structural heterogeneity in amorphous GBs (Figure 4b3) enables weak interfaces to facilitate atomic rearrangements for deformation accommodation.^56,57^ Unlike dislocation-STZ interactions in crystalline–amorphous nanocomposites, where activated STZs interact with adjacent crystals to trigger dislocation nucleation,^54,58^ interfacial STZs here percolate from weak interfaces to hard interiors within amorphous GBs, driving more uniform STZ distribution. These interior regions constrain STZ percolation, preventing shear band formation and strain concentration. Meanwhile, it is reported that the enhanced stability via GB amorphization can improve thermal stability,^35^ fracture toughness^59^ and ductility^22^ for crystalline–amorphous nanocomposites in the Cu–Zr system. From a composite theory perspective, GB amorphization effectively tunes the allocation of plastic deformation, mitigating detrimental dislocation-GB interactions while increasing the anticipation of stable and strong amorphous GBs. These findings provide a valuable framework for guiding experimental designs aimed at reinforcing NG metals. By adjusting solute concentrations and processing routes (e.g., thermal annealing), it is possible to precisely control the extent of GB amorphization, offering tunability to optimize mechanical properties, such as strength and ductility, to meet specific application needs.

Due to the significant GB volume fraction in extremely fine NG Cu, GB modifications substantially change deformation mechanisms, eventually affecting strength performance. As schematically illustrated in Figure 5a, continuous Zr segregation into pure NG Cu progressively modifies dominant deformation mechanisms. In pure NG Cu with extremely fine grains, GB migration, driven by repeated dislocation-GB interactions across entire GBs, causes significant strain concentrations, resulting in low deformation resistance. Introducing small amounts of Zr atoms to induce GB segregation can alleviate migration by reducing active dislocation sources and suppressing nucleation. However, GB regions with insufficient Zr segregation remain prone to migration. As the degree of segregation increases, it gradually leads to GB amorphization, forming nanometer-sized amorphous GBs characterized by structural heterogeneity between weak SRO-dominated interfacial regions and hard SRO-dominated interiors. As a result, the homogeneous STZ activation within amorphous GBs, coupled with significantly suppressed dislocation processes, dominates homogeneous deformation.

**Figure 5:**
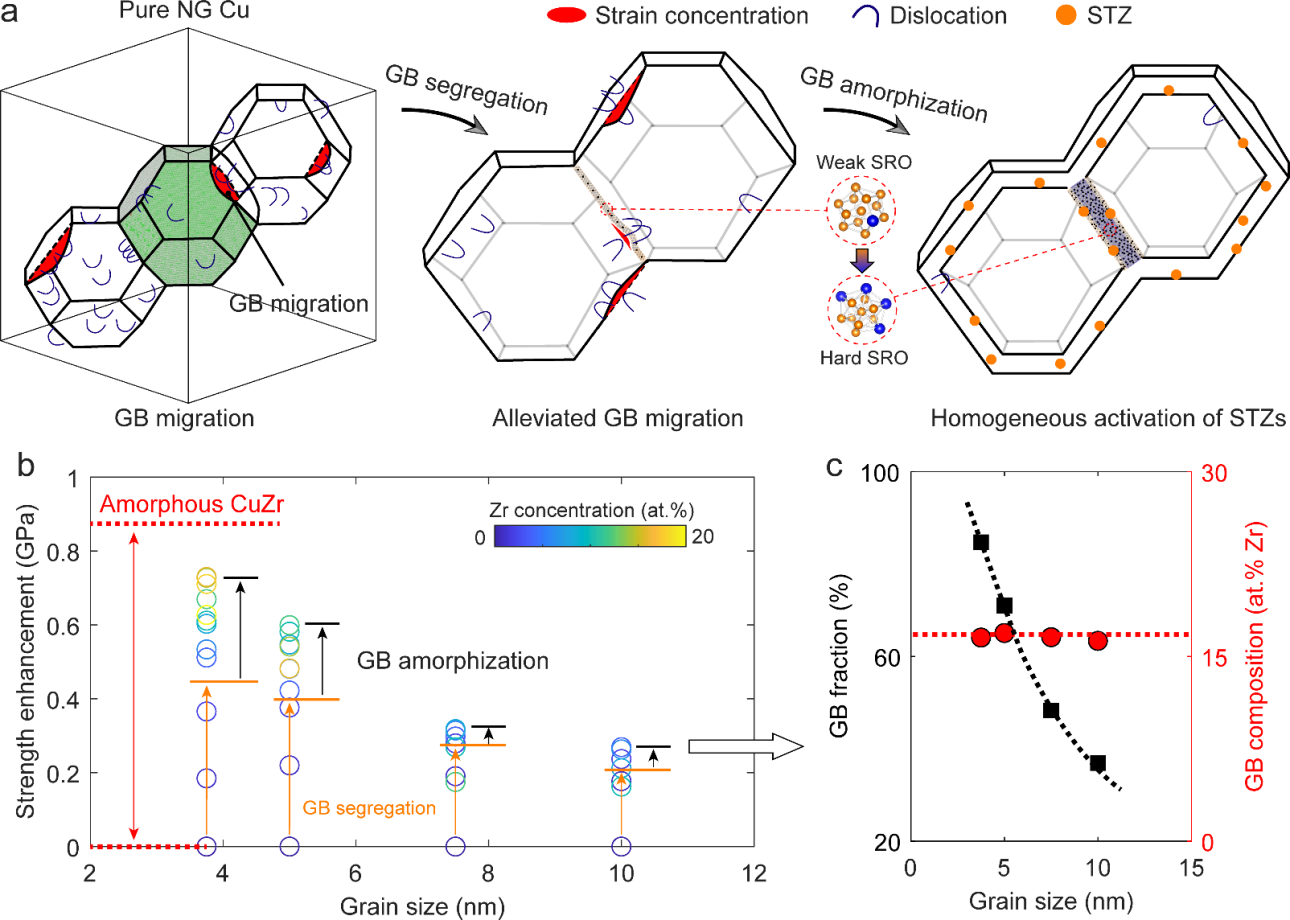
Extra strengthening effect resulting from GB amorphization. (a) Schematic illustration depicting the continuous transition in deformation mechanisms associated with GB segregationand GB amorphization. (b) Comparison of strength enhancement resulting from GB segregation versus GB amorphization, along with their dependencies on initial grain size and Zr concentration. The strength enhancement is calculated as the increase in strength of the Zr-segregated NG Cu sample compared to the pure NG Cu counterpart. The critical concentration to partition the strength effect raised from GB segregation or GB amorphization for each grain size is defined in Figure S6. (c) GB fraction and GB composition as a function of initial grain size for samples exhibiting maximum strength.

The strengthening effect of GB amorphization can be further amplified by refining grain size to several nanometers. As shown in Figure 5b, the strength enhancement from GB amorphization surpasses that from GB segregation, with more pronounced increases at smaller initial grain sizes. Following the fact of significant increase in volume fraction with decreasing grain size, GB amorphization-induced strengthening maximizes GB utilization to reinforce NG metals at smaller grain sizes, as indicated by the black dashed line in Figure 5c. Meanwhile, upon attaining the maximum strength, the GB composition remains nearly constant at approximately 17 at. % Zr for all extremely fine grain sizes, as indicated by the red dashed line. Given SRO fraction and average FFLS saturation at this local Zr concentration, it is reasonable to assume that amorphous GBs achieve their strongest state at this composition under selected simulation conditions (e.g., annealing temperature and cooling rate). For the smallest grain size of 3.75 nm, the strongest amorphous GB, with ∼17 at. % Zr and ∼85% volume fraction, achieves strength approaching that of nanosized amorphous Cu_80_Zr_20_ with similar composition. This suggests NG Cu with extremely fine grains and nanometer-sized amorphous GBs behaves similarly to nanosized amorphous counterparts, which theoretically undergo homogeneous deformation and exhibit ideal strength.^55^

While current research on GB solute segregation primarily focuses on preventing GB softening and suppressing dislocation activity, less attention has been given to GB amorphization strengthening, particularly in extremely fine NG metals. Experimental investigations may encounter significant challenges in preparing a series of samples with a fixed grain size within the extremely fine regime while simultaneously achieving controlled Zr concentration variations for property optimization.^22,38,39^ In this study, hybrid MC/MD simulations offer a practical approach, allowing precise control over grain size and solute concentration for desired sample preparation.^12,23,40,60^ Simulation findings provide insights into critical aspects such as individual and overall GB transition analysis, local GB structures (e.g., SROs), deformation mechanisms, and strength performance for Zr-segregated extremely fine NG Cu. Continuous Zr segregation leads to a progressive transition from original GBs, which undergo GB migration, to segregated GBs with alleviated migration, and eventually to amorphous GBs governed by homogeous STZ activation. GB amorphization-induced strengthening maximizes the utilization of high-density GBs to reinforce NG Cu with grain sizes on the order of several nanometers. This study represents an essential step toward deepening our understanding of GB segregation, amorphization, and their associate strengthening effects in extremely fine NG metals.

## Supporting Information Available

Texts: S1, material selection; S2, construction of atomic configurations; S3, uniaxial tensile test and visualization; S4, tensile behaviors of pure NG Cu; S5, uniform Zr segregation along GBs; S6, tensile behaviors of amorphous CuZr nanostructures; S7, McLean predication of local Zr concentration at GBs; S8, calculation of Voronoi index notation and five-fold local symmetry; S9, grain size/Zr concentration-dependent dislocation-GB interactions. Figures: S1, enthalpy of mixing for Cu–Zr system; S2, simulation methods; S3, tensile results of pure NG Cu and amorphous CuZr nanostructures; S4, GB misorientation; S5, local Zr concentration profiles; S6, average GB composition and GB thickness; S7, fractions of the ten most frequent SROs; S8, evolution of SRO fractions with increasing Zr concentration; S9, grain size/Zr concentration-dependent dislocation-GB interactions; S10, statistical analysis of dislocation line length; S11, statistical analysis of shear strain magnitude of GBs (PDF)
