# Supplementary material for: Maintaining Grain Boundary Segregation-Induced Strengthening Effect in Extremely Fine Nanograined Metals
Source: Nano Lett. 2025 Mar 24;25(13):5493–501. doi: 10.1021/acs.nanolett.5c01032 (PMC11969652; doi:10.1021/acs.nanolett.5c01032)
Supplement: Supplementary file 1 — nl5c01032_si_001.pdf [file nl5c01032_si_001.pdf]

Supporting Information for

Maintaining grain boundary segregation-induced strengthening effect in extremely fine nanograined metals

Lei Qian[a, b1], Jiacheng Zhang[a1], Wenqing Yang[a], Yunjiang Wang[c], K. C. Chan[a], and Xu-Sheng Yang[a, b]*

[a]Department of Industrial and Systems Engineering, Research Institute for Advanced Manufacturing, The Hong Kong Polytechnic University, Hung Hom, Kowloon, Hong Kong 999077, China

[b]Hong Kong Polytechnic University Shenzhen Research Institute, Shenzhen 518060, China

[c]State Key Laboratory of Nonlinear Mechanics, Institute of Mechanics, Chinese Academy of Sciences, Beijing 100080, China

[*]Corresponding authors.
E-mail address: xsyang@polyu.edu.hk (Xu-Sheng Yang), Tel: +852-27666604
[1]These authors contributed equally to this work

This file includes:

Text S1. Material selection

Text S2. Construction of atomic configurations

Text S3. Uniaxial tensile test and visualization

Text S4. Tensile behaviors of pure NG Cu

Text S5. Uniform Zr segregation along GBs

Text S6. Tensile behaviors of amorphous CuZr nanostructures

Text S7. McLean predication of local Zr concentration at GBs

Text S8. Calculation of Voronoi index notation and five-fold local symmetry

Text S9. Grain size/Zr concentration-dependent dislocation-GB interactions

Figure S1. Enthalpy of mixing for Cu-Zr system

Figure S2. Simulation methods

Figure S3. Tensile results of pure NG Cu and amorphous CuZr nanostructures

Figure S4. GB misorientation

Figure S5. Local Zr concentration profiles

Figure S6. Average GB composition and GB thickness

Figure S7. Fractions of the ten most frequent SROs

Figure S8. Evolution of SRO fractions with increasing Zr concentration

Figure S9. Grain size/Zr concentration-dependent dislocation-GB interactions

Figure S10. Statistical analysis of dislocation line length

Figure S11 Statistical analysis of shear strain magnitude of GBs

Text S1. Material selection

To investigate the intentional grain boundary (GB) segregation behavior and consequent strengthening effect in extremely fine nanograined (NG) metals, hybrid Monte Carlo/Molecular Dynamics (MC/MD) simulations were performed with the Large-scale Atomic/Molecular Massively Parallel Simulator (LAMMPS) software [1]. The Cu-Zr alloy system, widely recognized as a model for studying segregation-induced GB transitions and their impact on mechanical properties [2,3], was selected as an optimal candidate for exploring GB segregation-induced strengthening effects in extremely fine NG Cu. Zr atoms, due to their significant atomic size mismatch relative to Cu and extremely low solubility (~ 0.12 at. % at the eutectic temperature [4,5]), preferentially segregate to GBs, a behavior confirmed by their positive enthalpy of segregation [6]. Additionally, the Cu-Zr system is characterized by a negative enthalpy of mixing across a wide compositional range [7,8], indicating its exceptional glass forming ability, as also verified by our simulation results (see Figure S1). This combination of positive enthalpy of segregation and negative enthalpy of mixing aligns with established material selection criteria for the formation of amorphous GBs within a crystalline matrix [9,10]. Under appropriate thermal and compositional conditions, Zr segregation enriches GBs in Cu, often leading to GB amorphization, which has been extensively documented through both experimental and simulation-based studies [2,3,11–14]. For example, experimental study on Zr doping in Cu have reported the emergence of the well-defined segregation at GBs at low Zr concentration and the eventual formation of continuous amorphous GB networks at relatively high Zr concentration (~7.0 at.% Zr)[11]. These features establish the Cu-Zr system as an ideal platform for investigating the unexplored topic of strengthening effects induced by GB

amorphization in NG Cu with extremely fine grain sizes. To accurately model atomistic interactions within the Cu-Zr system, the embedded-atom method (EAM) interatomic potential was employed [15], which has been validated in previous studies focused on solute-GB interactions.

Text S2. Construction of atomic configurations

Using a Voronoi tessellation approach, a three-dimensional simulation box was divided into sixteen randomly oriented Cu nanograins with truncated-octahedron geometries [16,17] and face-centered-cubic (fcc) lattice structures, as depicted in Figure S2a. The generated atomic configurations are consistent with Kelvin crystals (KCs), consisting of sixteen Kelvin cells with uniform edge length l (Figure S2b) [16,17]. The grain size d, representing the characteristic geometric dimension of the nanograin (also corresponding to the Kelvin cell), was determined as $d = 3l$ [18]. By scaling the edge length to change the dimensions of atomic configurations, NG samples with grain sizes ranging from 3.75 nm to 30 nm were constructed.

The constructed pure NG Cu samples were then doped with Zr atoms at varying concentrations using the hybrid MC/MD method to simulate GB segregation, following the approach outlined in previous studies [19,20], as illustrated in Figure S2d. Specifically, Zr atoms were introduced by replacing a fraction of Cu atoms within the MC swap regions near GBs, as shown in Figure 1a, enabling controlled segregation behavior under predefined thermodynamic and kinetic conditions. The total Zr concentration, reaching up to 20 at. %, was precisely controlled by adjusting the fraction of Cu atoms replaced in the MC swap region relative to the total number of atoms in the atomic configuration of the sample. To achieve equilibrium, the doped samples were equilibrated under isothermal-isobaric (NPT) conditions at 300 K and zero

applied stress until the system's potential energy converged. It was run for up to 10^5 MD steps and calls to MC every 10 MD steps. The precise control over solute concentration and equilibrium solute distributions achieved in this process ensure the accurate modeling of GB segregation behavior, which is critical for subsequently investigating GB structural transitions and their effects on mechanical response [19,20]. After segregation to the desired concentration, the sample was annealed at 800 K and zero applied stress for 1 ns, followed by rapid cooling at $5 \times 10^{12}\ K\ s^{-1}$ to 300 K and equilibrating at this temperature for another 100 ps, as shown in Figure S2d. The glass transition temperatures for Cu-Zr systems with Zr concentrations ranging from 10 at. % to 50 at. % were calculated to be approximately 700 K, as shown in Figure S2e. Annealing at 800 K, which is slightly above the glass transition temperature, coupled with rapid cooling, promotes the amorphization of Zr-enriched regions and facilitates the formation of uniform nanometer-sized amorphous GBs in NG Cu samples [13,21,22]. In contrast, as-built pure NG Cu samples were relaxed at 300 K and zero applied stress to reach equilibrium. Additionally, a series of $Cu_{80}Zr_{20}$ metallic glass nanostructures with dimensions comparable to NG Cu samples with a grain size of 7.5 nm were prepared by heating to 2000 K to ensure complete melting, followed by rapid cooling to 300 K at varying cooling rates, as shown in Figure S2c. The heating temperature of 2000 K aligns with prior MD studies of Cu-Zr glass formation [23,24], while the relatively high cooling rates are consistent with MD timescale limitations and protocols proven to inhibit crystallization in Cu-Zr system [23,25]. All simulations employed an NPT ensemble with a timestep of 2 fs.

Text S3. Uniaxial tensile test and visualization

Equilibrium samples were subjected to uniaxial tensile deformation by stretching the simulation box along the z-direction at an engineering strain rate of 10^9 s^{-1} until 8% strain. To maintain uniaxial tensile conditions, the NPT ensemble was employed at 300 K, with zero pressure applied along the x and y directions, and periodic boundary conditions were implemented in all directions. Due to computational limitations, MD simulations typically utilize strain rates significantly higher than those used in experiments. This approach ensures that mechanical testing and deformation behavior can be observed within nanosecond timescales and at nanometer length scales. Despite these constraints, MD simulations are widely recognized for their ability to accurately capture deformation mechanisms and strength trends in nanostructured metals, including Cu and Cu-based alloys [17,26–28].

As MD simulations normally start from constructed samples with few dislocations inside nanograins, a large stress is required to activate dislocation nucleation from sources of GBs, showing an initial peak of flow stress in the stress-strain curve. With the increasing of tensile strain, nucleated dislocations propagated inside grains and massive plastic deformation occurs, leading to a gradual drop of flow stress. The seemingly softening and unstable phenomenon was commonly observed in MD simulations for nanostructured metals and known as the stress overshoot [29]. To better evaluate the strength performance of the simulated samples, the strength was calculated as the average stress between 6% and 8% strain, following established practices in MD simulations to minimize the influence of stress overshoot and to provide a stable and representative metric for strength performance [26,27]. While the absolute strength values obtained in MD simulations may differ from experimental results, the relative trends and mechanistic insights derived from simulations are critical for understanding segregation-

induced strengthening effects. All atomic configurations were visualized using the open-source visualization tool OVITO [30]. Atoms were colored based on their local crystal structure as identified by adaptive common neighbor analysis (CNA): fcc atoms are shown in green, hcp atoms in red, bcc atoms in blue, and disordered atoms (e.g., GBs and dislocation cores) in white. Information on dislocation line length was extracted from the MD trajectories using the dislocation extraction algorithm (DXA) in OVITO software.

Text S4. Tensile results of pure NG Cu

Figure S3a,b shows the tensile behaviors of pure NG Cu, revealing a grain size-dependent transition from Hall-Petch strengthening to inverse Hall-Petch softening, with a maximum strength of ~2.4 GPa at a grain size of d = 12.5 nm. As demonstrated in Figure S3b, when the grain size enters the softening regime of several nanometers, the volume fraction of GBs increases significantly. The contribution of GB atoms and the grain interior (GI) atoms to overall deformation can be quantitatively assessed through strain accommodation ratio (SAR) [31]. Figure S3c illustrates the inverse relationship between the fractions of strain accommodated by GB and GI atoms with decreasing grain size. A crossover is observed within the 10-15 nm grain size range, corresponding to the point where flow stress reaches its maximum. This crossover indicates a shift in the dominant deformation mechanism, transitioning from GI-based intragranular processes to GB-based intergranular processes as the grain size decreases.

Text S5. Uniform Zr segregation along GBs

Experimental works have demonstrated diverse behaviors of Zr doping in Cu, including homogeneous segregation along GBs [2], cluster formation [11], and GB amorphization [32], depending on the processing route and composition [3]. These observations highlight the critical

importance of understanding and controlling solute-induced GB transitions as a strategy to tailor the mechanical properties of NG metals, particularly those with extremely fine grain sizes [33,34]. In our simulations, the intentional GB segregation method enabled the initially homogeneous distribution of Zr atoms within interfacial regions between adjacent nanograins [19,20]. To quantify GB misorientation, defined as the disorientation angle between neighboring nanograins [22,35], we utilized the Grain Tracking Algorithm (GTA) [36]. This algorithm accurately identifies crystallographic characteristics, including GB misorientation, for simulated samples by using a strict misorientation cutoff angle of 1°. As shown in Figure S4a, the misorientation angles for various GBs range from 5° to 60° and exhibit negligible changes after Zr segregation, covering nearly the entire range expected for polycrystalline samples with randomly oriented nanograins [37,38]. Moreover, a significant fraction of high-angle GBs (misorientation angles >15°) was observed in Figure S4b, which are typically associated with elevated GB energies and a strong tendency for solute segregation [39,40]. The combination of the initial homogeneous Zr distribution within interfacial regions and high-angle GBs promotes uniform Zr segregation along GBs, potentially leading to the formation of three-dimensional (3D) GB networks with structural and compositional transitions.

Text S6. Tensile results of amorphous CuZr nanostructure

To compare the strength performance of Zr-segregated extremely fine NG Cu with its amorphous counterpart, a series of $Cu_{80}Zr_{20}$ metallic glass nanostructures with dimensions comparable to NG Cu samples with a grain size of 7.5 nm were prepared. Given the significant influence of cooling rate on the strength of Cu-Zr metallic glasses [41], a wide range of cooling rate ranging from $10^9\ K\ s^{-1}$ to $10^{13}\ K\ s^{-1}$ was employed to obtain a more representative

assessment of their strength performance. As plotted in Figure S3d, tensile results of amorphous $Cu_{80}Zr_{20}$ nanostructures reveal an increase in strength with increasing cooling rate. The highest strength observed at a cooling rate of $10^9\ K\ s^{-1}$ was used to represent the strength of amorphous $Cu_{80}Zr_{20}$ nanostructures.

Text S7. McLean predication of local Zr concentration at GBs

In a polycrystalline system, the total solute concentration X^{tot} is partitioned between the GI and the GB with the average solute contents denoted as X^c and X^{GB}, respectively, which could be expressed as [42]:

$$X^{tot} = (1 - f_{GB})X^c + f_{GB}X^{GB} \tag{1}$$

where f_{GB} is the fraction of atoms in GB region and typically associated with grain size (*d*) and thickness (*t*) using $f_{GB} = 1 - [(d - t)/d]^3$ [33]. Assuming ideal solution behavior within both the GB and GI, the equilibrium GB segregation state can be modeled using the classical McLean isotherm [43]. The average solute concentration at the GB ($\bar{X}^{GB}$) is given by:

$$\bar{X}^{GB} = \left[1 + \frac{1-X^c}{X^c} exp\left(-\frac{\Delta\bar{E}^{seg}}{kT}\right)\right]^{-1} \tag{2}$$

where T is the temperature, and k is the Boltzmann constant, and $\Delta\bar{E}^{seg}$ represents the segregation energy, defined as the energy difference between a solute atom occupying a GB site (E_{GB}^{solute}) and a bulk site (E_c^{solute}): $\Delta\bar{E}^{seg} = E_{GB}^{solute} - E_c^{solute}$. This energy difference serves as a measure of the solute's tendency to segregate to the GB. Following McLean's treatment, $\Delta\bar{E}^{seg}$ is assumed to be a single value for all GB types, neglecting variations in GB characters and solute-solute interactions. For example, using $f_{GB} = 24\%$ for pure NG Cu with constant d = 7.5 nm, t = 0.65 nm and $\Delta\bar{E}^{seg} = 0.3\ eV$ (a reasonable averaged value

representing strong Zr segregation to GBs) [44,45], the McLean model predicts local Zr concentrations at GBs that match well with simulation results up to a total solute concentration of ~ 2 at. % for grain size of 7.5 nm, as plotted by the orange dashed line in Figure S6b. Beyond this concentration, however, significant deviations are observed, indicating the formation of distinct GBs. This deviation primarily results from continuous segregation-induced changes in GB structure and composition, which modify the GB fraction (f_{GB}) in Eq. (1) or alter the segregation energy ($\Delta\bar{E}^{seg}$) in Eq. (2) [10,46]. From the perspective of GB fraction (f_{GB}), we consider the observed GB thickening at a higher total solute concentration, which reallocates the fraction between the GB and GI atoms, thereby altering the local concentration. For instance, at a total Zr concentration of 10 at. %, accounting for GB thickening (t = 1.9 nm) in the McLean model predicts a local Zr concentration of 20.6 at. %, closely matching the simulation result of 19.3 at. %. The intentional GB segregation eventually results in a ~ 2 nm thickness GB with ~20 at. % local Zr concentration at a total concentration of approximately 10 at. %, distinguishing these GBs from original or segregated GBs. The onset of deviation and GB thickening thus define a critical solute concentration (indicated by the dot-dash line in Figure S6), which indicates the saturation state of segregated GBs and the start to induce distinct GBs, namely amorphous GBs. The combined approach of analyzing both individual GBs and overall GB networks provides a more accurate determination of the critical Zr concentration for inducing GB amorphization. Additionally, the critical concentration varies with grain size, typically falling within a total concentration range of 1-3 at. % for grain sizes ranging from 3.75 nm to 10 nm. While the critical concentration exhibits slight variations depending on grain size, the transition to segregation-induced GB amorphization due to GB

thickening and local Zr enrichment remains consistent across all extremely fine grain sizes studied.

Text S8. Calculation of Voronoi index notation and five-fold local symmetry

Considering the individual GB as a repetition and connection of different local atomic structures, GBs can be evaluated through the identification of local structural units, like SROs [47]. Following the proposed approach [48], all local structures within the GB regions in obtained equilibrium samples were identified using the Voronoi tessellation method and characterized as Voronoi polyhedrons associated with each atoms [49,50]. Each polyhedron is described using Voronoi index notation $\langle n_3, n_4, n_5, n_6 \rangle$, where n_i stands for the number of Voronoi polyhedron faces with i edges. The fraction of specific Voronoi polyhedron type describes the preferred SRO in the GB regions. As shown in Figure S7, at low concentrations (≤ 2 at. %), the two dominant SRO types are <0, 10, 2, 0> and <0, 8, 4, 0> with relatively ordered structures, which are distorted versions of the perfect fcc polyhedron <0, 12, 0, 0> [51]. However, at a Zr concentration of 12 at. %, more distorted polyhedron, such as <0, 2, 10, 0>, <0, 4, 8, 0> and <0, 0, 12, 0>, predominate. The full-icosahedral cluster, <0, 0, 12, 0>, is a common five-fold symmetric quasi-crystalline packing motif in amorphous structures or metallic glasses [52,53], suggesting that the local atomic arrangements of highly segregated GBs resemble those of amorphous phases.

Upon obtaining the Voronoi index notation for each atom via the Voronoi tessellation method, the average degree of k-fold local symmetry can be defined as $\sum_i \left(f_i^k \times P_i\right)$, where P_i is the fraction of polyhedron type i and f_i^k represents the fraction of k-edged polygon in

Voronoi polyhedron type i and is defined as $f_i^k = n_i^k / \sum_{k=3,4,5,6} n_i^k$. Here, n_i^k denotes the number of k-edged polygon in Voronoi polyhedron type i. Thus, the average FFLS can be calculated as follows [23],

$$FFLS = \sum_i \left(f_i^5 \times P_i \right). \quad (3)$$

Text S9. Grain size/Zr concentration-dependent dislocation-GB interactions

In the conventional Hall-Petch strengthening regime (here, d ≥ 12.5 nm), the dislocation-mediated plasticity dominates deformation, where Shockley partial dislocations (indicated by green lines) nucleated from GBs (indicated by grey interfaces) propagate across the nanograins with intragranular motion, as shown in Figure S9a. However, as the nanograin is refined into the softening regime (here, d < 12.5 nm), the dislocation activity in pure NG Cu behaves preferentially like dislocations occurring near or within GBs, as indicated by localized dislocation segments near GBs in Figure S9b. When segregating Zr into extremely fine NG Cu and increasing the total Zr concentration, the pre-existing dislocations in undeformed samples and newly formed dislocations in deformed samples deceases across all studied grain sizes. Experimental and simulation study has demonstrated the presence of dislocations within or near GB regions in extremely fine NG metals [54]. Meanwhile, previous studies have linked pre-existing GB dislocations with GB softening mechanisms, such as GB migration, sliding and rotation, which may contribute to strength softening [55–59]. This highlights the importance of dislocation-GB interactions in determining deformation mechanisms and resultant strength performance.

Figure S1. Enthalpy of mixing for Cu-Zr system

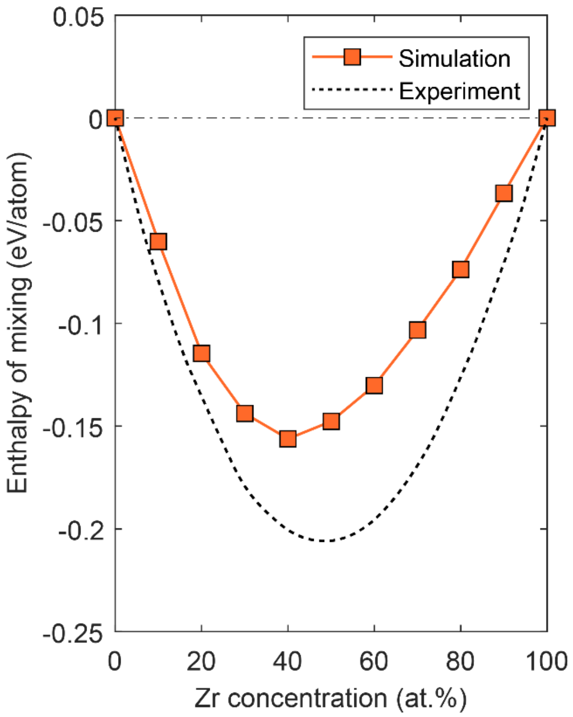

Figure S1. Enthalpy of mixing as a function of total Zr concentration for Cu-Zr system at 1500 K. The experiment data were obtained from reference [60].

Figure S2. Simulation methods

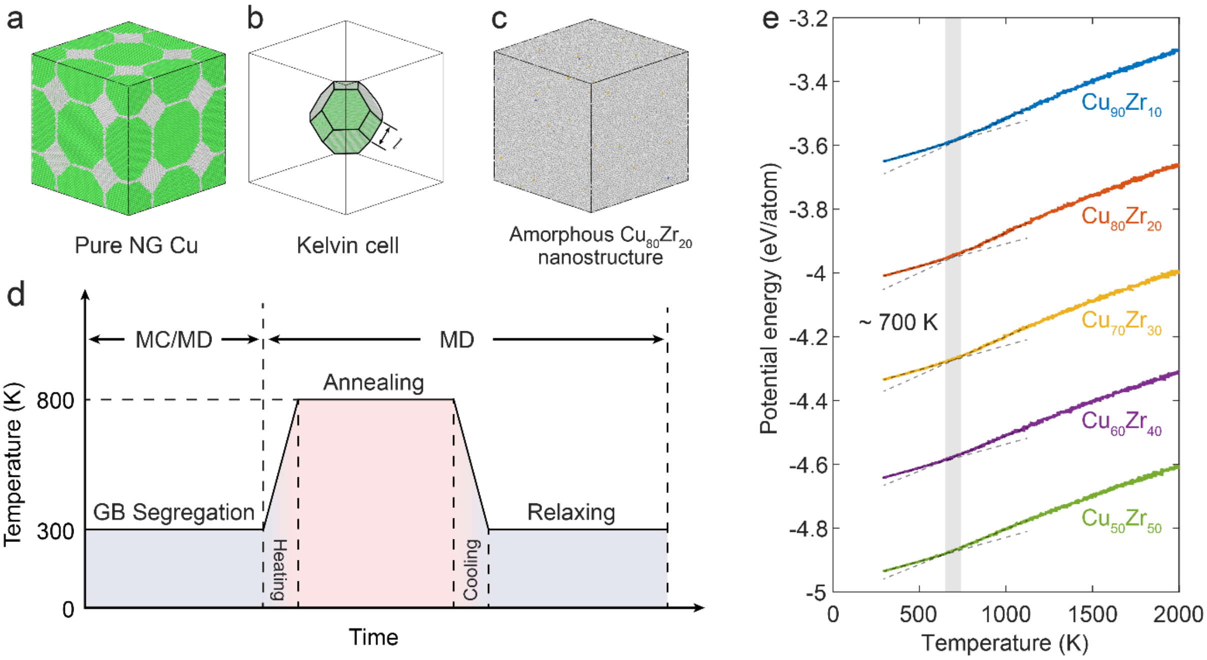

Figure 2. Simulation methods. (a) Atomic configuration of the constructed pure NG Cu sample with a grain size of *d*. (b) Atomic configuration of the kelvin cell with an edge length of *l*. (c) Atomic configuration of the prepared amorphous $Cu_{80}Zr_{20}$ nanostructure. (d) Illustration of Hybrid MC/MD simulation, following steps of GB segregation, annealing and relaxing. (e) Potential energy as a function of temperature to determine the glass transition temperature for Cu-Zr system with Zr concentration ranging from 10 at. % to 50 at. %. The calculated glass transition temperature is ~700 K, in good agreement with experiments and simulations [61,62].

Figure S3. Tensile results of pure NG Cu and amorphous CuZr nanostructures

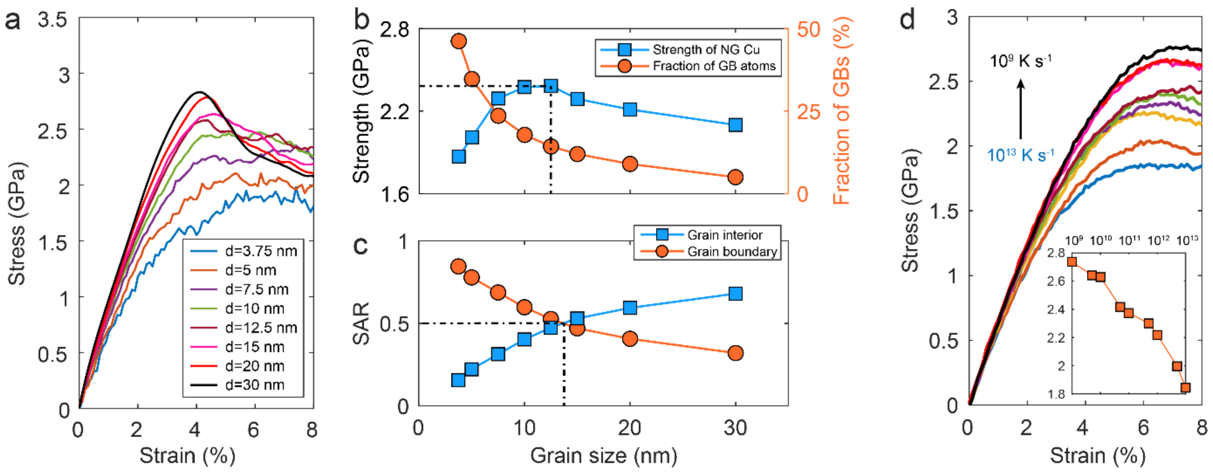

Figure S3. Tensile results of pure NG Cu and amorphous $Cu_{80}Zr_{20}$ nanostructures. (a) Stress-strain curves of pure NG Cu. (b) Variations of strength and GB fraction as a function of grain size for pure NG Cu. (c) Variations of SAR of GB atoms and GI atoms as a function of grain size at 8% strain. (d) Stress-strain curves for amorphous $Cu_{80}Zr_{20}$ nanostructures prepared under cooling rates ranging from $1 \times 10^{9}\ K\ s^{-1}$ to $1 \times 10^{13}\ K\ s^{-1}$. The inserted figure in (d) shows variations of calculated strength as a function of cooling rate.

Figure S4. GB misorientation

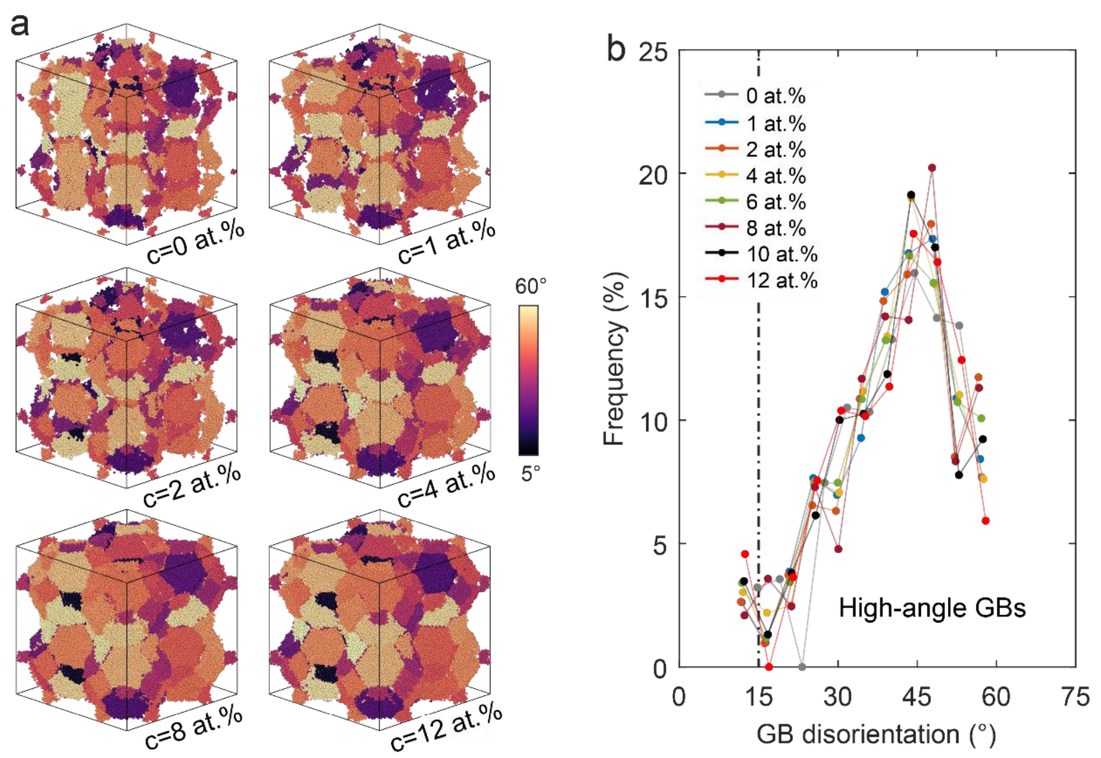

Figure S4. GB misorientation in NG Cu with a grain size of 7.5 nm. (a) Distribution of disorientation angles between adjacent nanograins, representing the GB misorientation. Atoms at triple junctions are excluded for clarity. (b) GB fraction as a function of GB disorientation angle in samples with varying total Zr concentrations, showing a great proportion of high-angle GBs (i.e., > 15°).

Figure S5. Local Zr concentration profiles

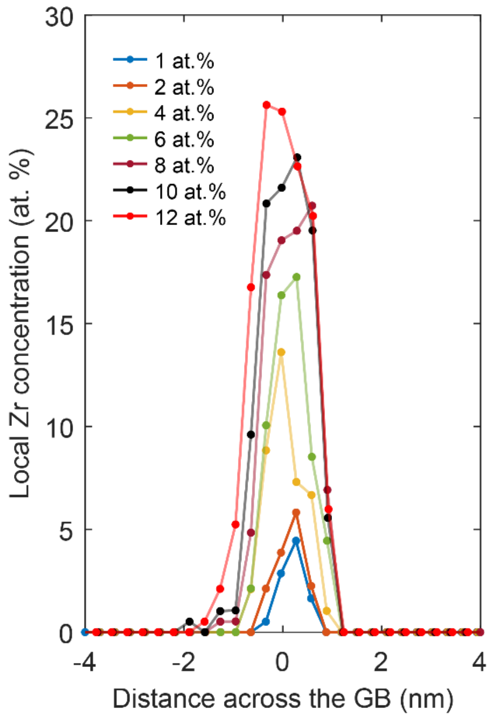

Figure S5. Variations of local Zr concentration profiles across representative bicystal regions for varying total Zr concentrations.

Figure S6. Average GB composition and GB thickness

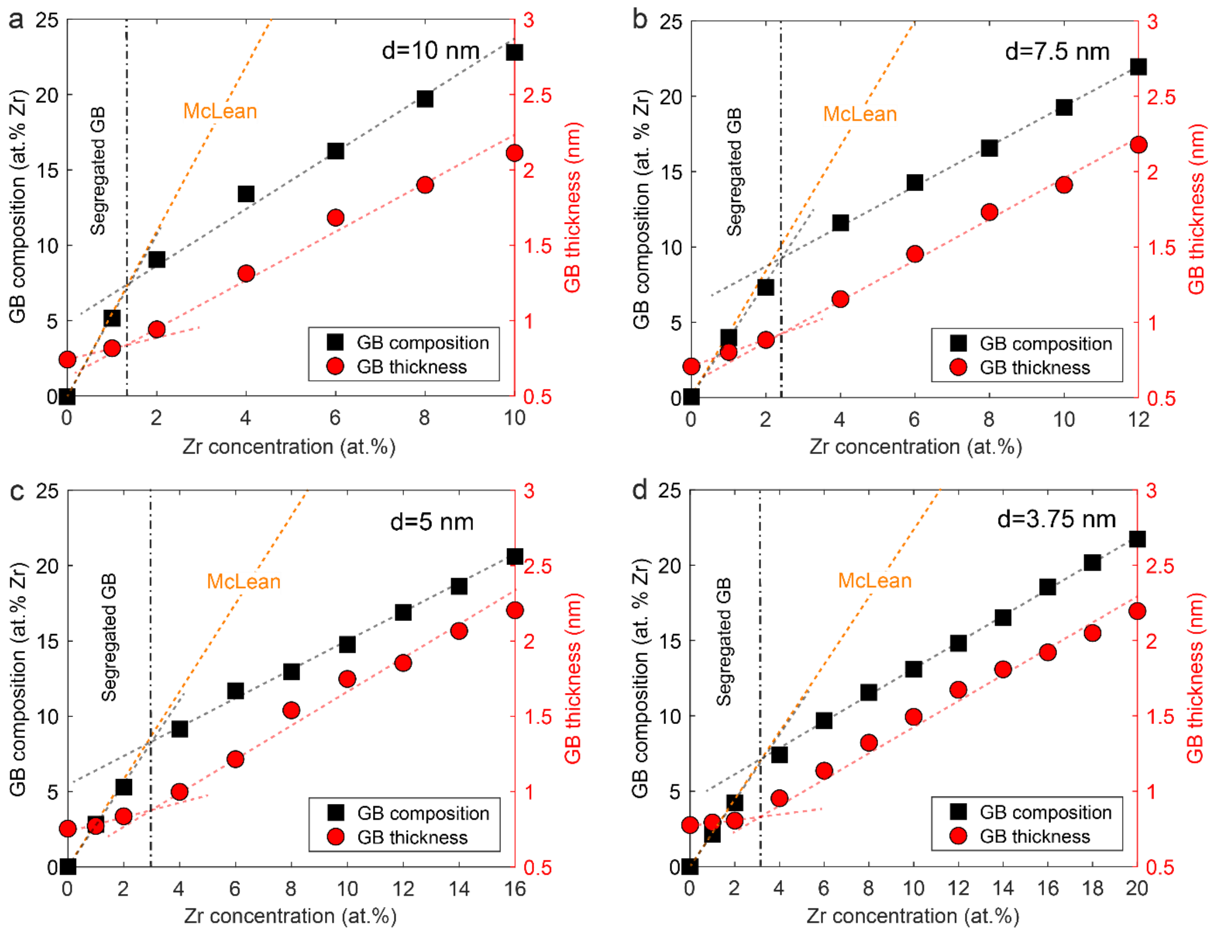

Figure S6. Average GB composition and GB thickness as a function of total Zr concentration in NG Cu with grain size of (a) 10 nm, (b) 7.5 nm (c) 5 nm and (d) 3.75nm. The dot-dash lines indicate the critical Zr concentration for onset of GB amorphization

Figure S7. Fractions of the ten most frequent SROs

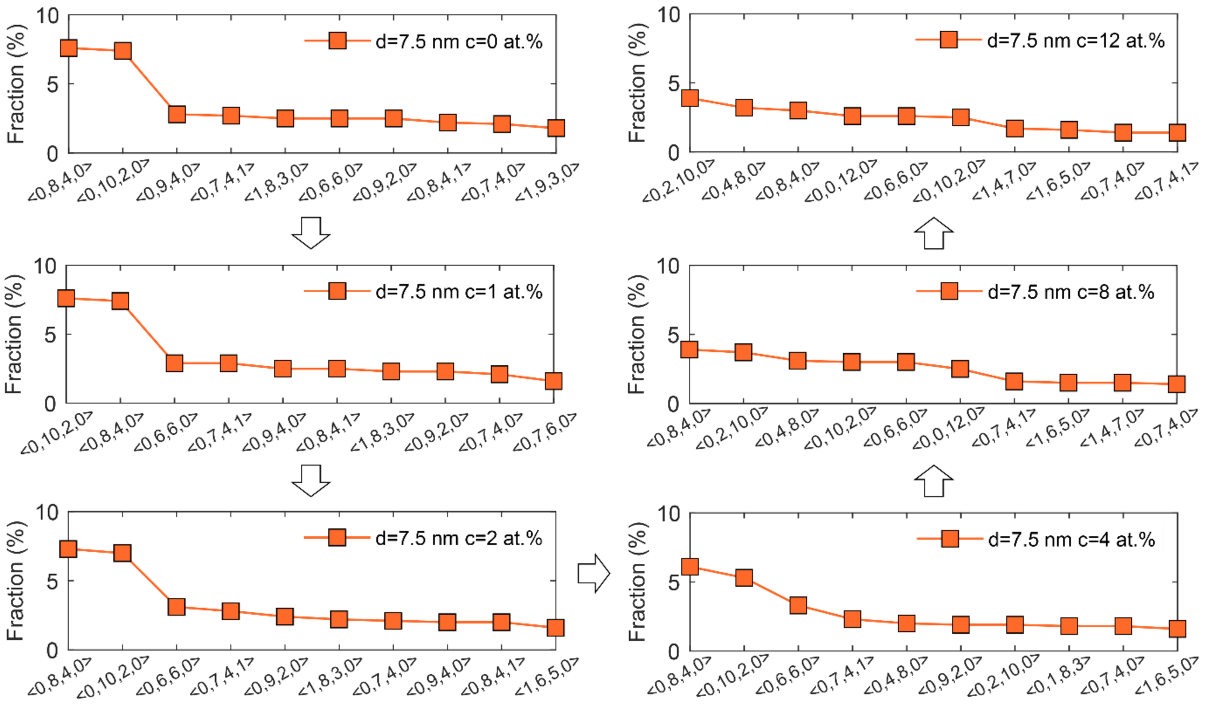

Figure S7. Calculated fractions of atoms associated with the ten most frequent SROs across different Zr concentrations in NG Cu with grain size of 7.5 nm.

Figure S8. Evolution of SRO fractions with increasing Zr concentration

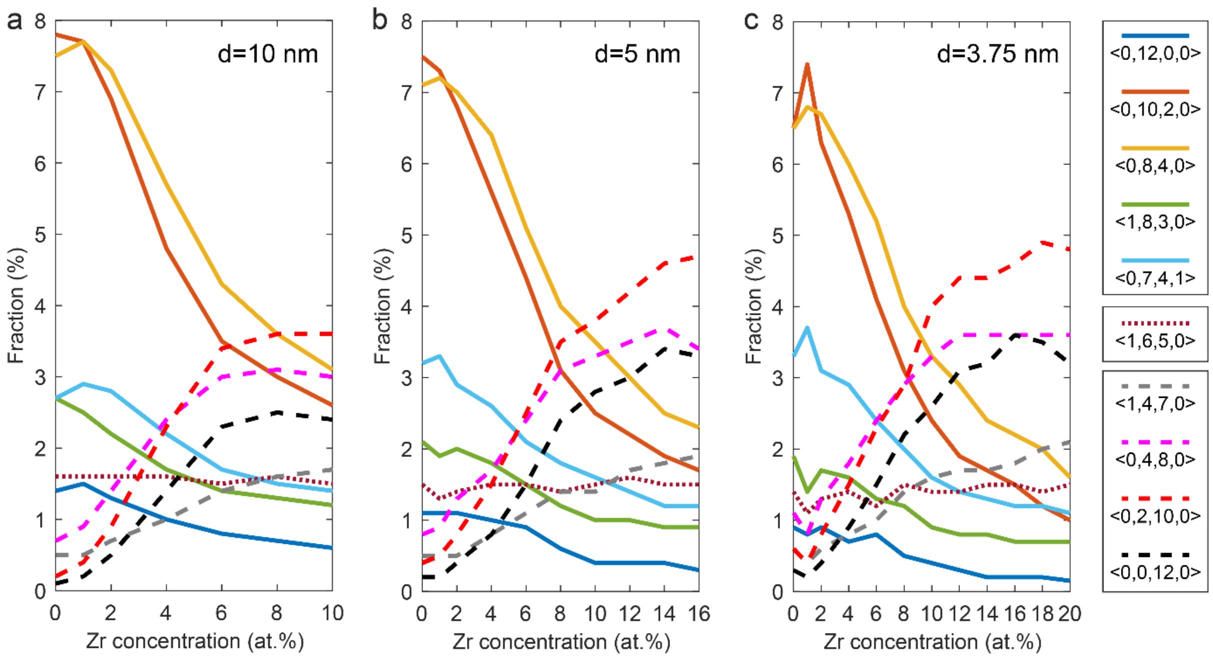

Figure S8. Evolution of fractions of representative SROs with increasing Zr concentration for grain size of (a) 10 nm, (b) 5 nm and (c) 3.75 nm.

Figure S9. Grain size/Zr concentration-dependent dislocation-GB interactions

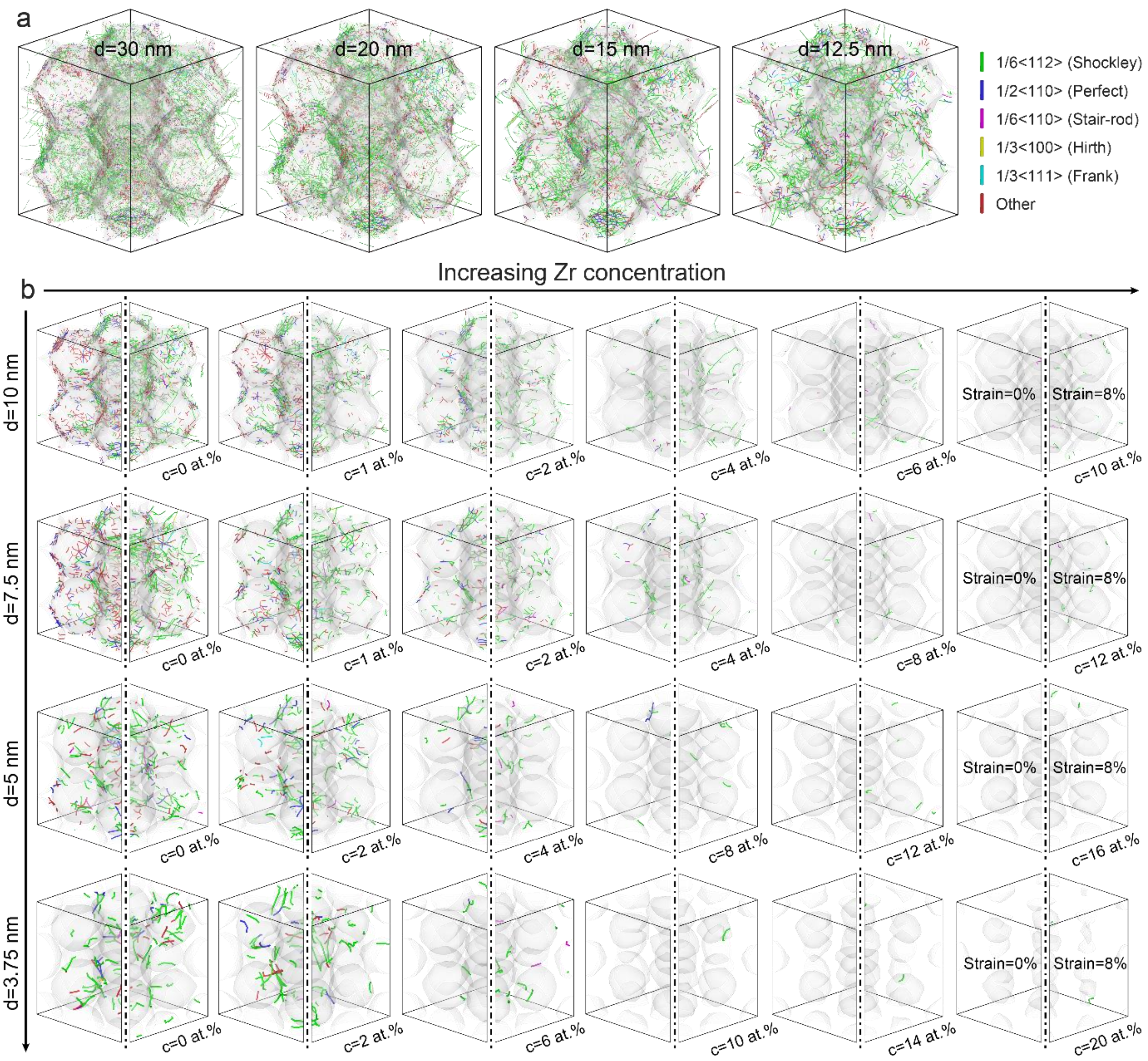

Figure S9. Grain size/Zr concentration-dependent dislocation-GB interactions in NG Cu. (a) Dislocation distribution in pure NG Cu samples with various grain sizes after deformation (at 8% strain). (b) Dislocation distributions in undeformed samples at 0% strain (left part of each subfigure) and deformed samples at 8% strain (right part of each subfigure) as functions of grain size and Zr concentration. Only reconstructed GBs in grey color and dislocation lines are displayed for clarity.

Figure S10. Statistical analysis of dislocation line length

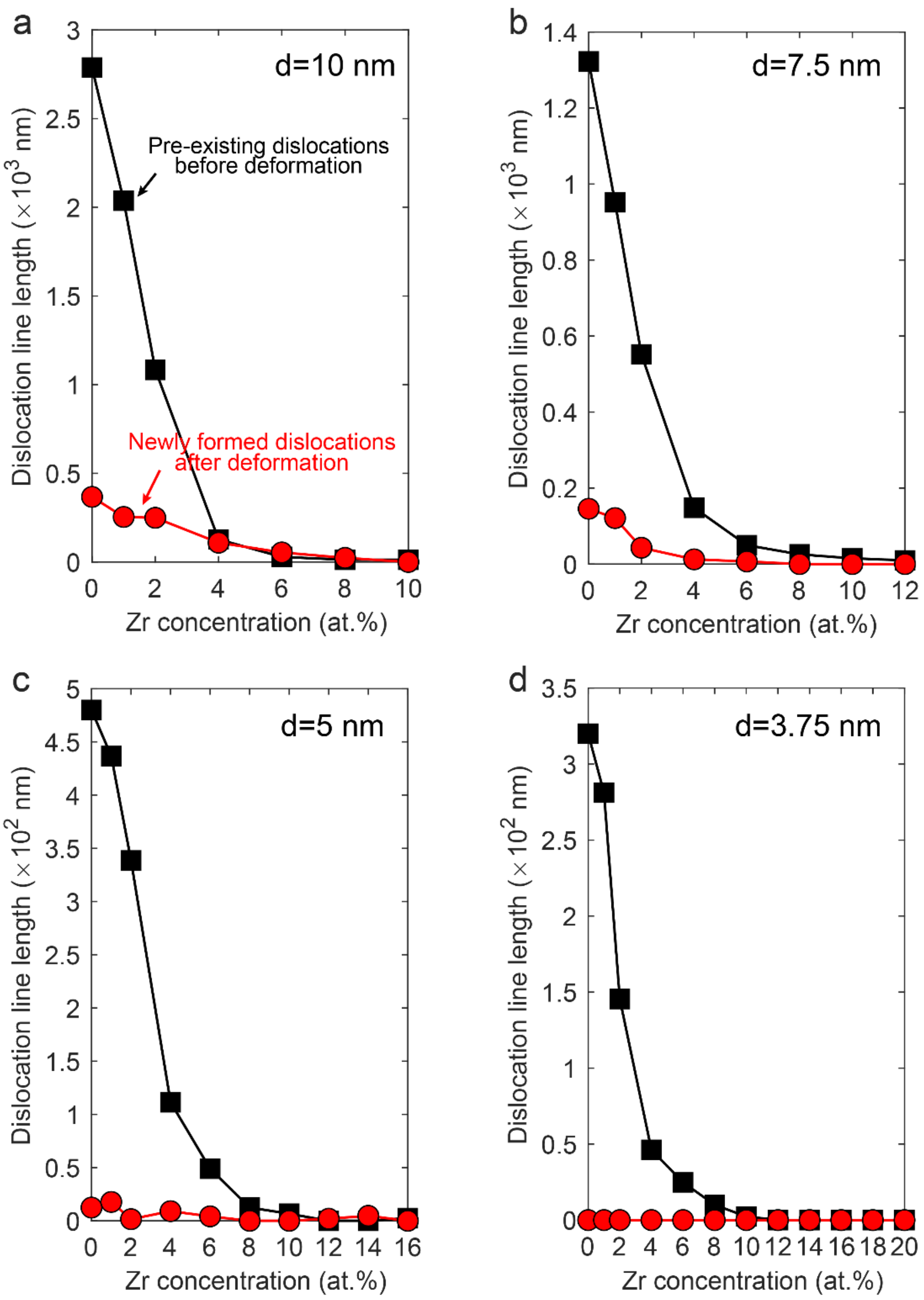

Figure S10. Evolution of dislocation line length with increasing total Zr concentration for grain size of (a) 10 nm, (b) 7.5 nm (c) 5 nm and (d) 3.75nm.

Figure S11. Statistical analysis of shear strain magnitude of GBs

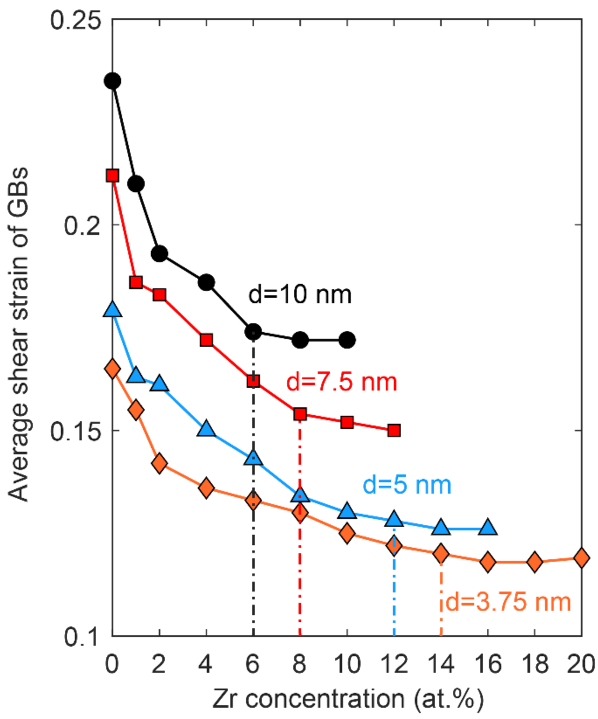

Figure S11. Evolution of average shear strain magnitude of GBs at 8% strain with increasing total Zr concentration for studied grain sizes ranging from 3.75 nm to 10 nm. The dot-dash lines indicate the critical Zr concentration required to form fully amorphous GBs.

References

(1) Thompson, A. P.; Aktulga, H. M.; Berger, R.; Bolintineanu, D. S.; Brown, W. M.; Crozier, P. S.; Veld, P. J. in 't; Kohlmeyer, A.; Moore, S. G.; Nguyen, T. D.; Shan, R.; Stevens, M. J.; Tranchida, J.; Trott, C.; Plimpton, S. J. LAMMPS - a flexible simulation tool for particle-based materials modeling at the atomic, meso, and continuum scales. *Comput. Phys. Commun.* **2022**, *271*, 108171.

(2) Meiners, T.; Duarte, J. M.; Richter, G.; Dehm, G.; Liebscher, C. H. Tantalum and zirconium induced structural transitions at complex [111] tilt grain boundaries in copper. *Acta Mater.* **2020**, *190*, 93–104.

(3) Khalajhedayati, A.; Pan, Z.; Rupert, T. J. Manipulating the interfacial structure of nanomaterials to achieve a unique combination of strength and ductility. *Nat. Commun.* **2016**, *7*, 10802.

(4) Arias, D.; Abriata, J. P. Cu-Zr (copper-zirconium). *Journal of Phase Equilibria* **1990**, *11* (5), 452–459.

(5) Atwater, M. A.; Scattergood, R. O.; Koch, C. C. The stabilization of nanocrystalline copper by zirconium. *Mater. Sci. Eng., A* **2013**, *559*, 250–256.

(6) Murdoch, H. A.; Schuh, C. A. Estimation of grain boundary segregation enthalpy and its role in stable nanocrystalline alloy design. *J. Mater. Res.* **2013**, *28* (16), 2154–2163.

(7) Xu, D.; Lohwongwatana, B.; Duan, G.; Johnson, W. L.; Garland, C. Bulk metallic glass formation in binary Cu-rich alloy series – Cu100−xZrx (x=34, 36, 38.2, 40 at.%) and mechanical properties of bulk Cu64Zr36 glass. *Acta Mater.* **2004**, *52* (9), 2621–2624.

(8) Turchanin, M. A. Calorimetric research on the heat of formation of liquid alloys of copper with group IIIA and group IVA metals. *Powder Metallurgy and Metal Ceramics* **1997**, *36* (5), 253–263.

(9) Schuler, J. D.; Rupert, T. J. Materials selection rules for amorphous complexion formation in binary metallic alloys. *Acta Mater.* **2017**, *140*, 196–205.

(10) Hu, C.; Dingreville, R.; Boyce, B. L. Computational modeling of grain boundary segregation: A review. *Comput. Mater. Sci.* **2024**, *232*, 112596.

(11) Zhao, J. T.; Zhang, J. Y.; Cao, L. F.; Wang, Y. Q.; Zhang, P.; Wu, K.; Liu, G.; Sun, J. Zr alloying effect on the microstructure evolution and plastic deformation of nanostructured Cu thin films. *Acta Mater.* **2017**, *132*, 550–564.

(12) Khalajhedayati, A.; Rupert, T. J. High-Temperature Stability and Grain Boundary Complexion Formation in a Nanocrystalline Cu-Zr Alloy. *JOM* **2015**, *67* (12), 2788–2801.

(13) Pan, Z.; Rupert, T. J. Effect of grain boundary character on segregation-induced structural transitions. *Phys. Rev. B* **2016**, *93* (13).

(14) Zhang, P.; Zhang, J. Y.; Li, J.; Liu, G.; Wu, K.; Wang, Y. Q.; Sun, J. Microstructural evolution, mechanical properties and deformation mechanisms of nanocrystalline Cu thin films alloyed with Zr. *Acta Mater.* **2014**, *76*, 221–237.

(15) Borovikov, V.; Mendelev, M. I.; King, A. H. Effects of stable and unstable stacking fault energy on dislocation nucleation in nano-crystalline metals. *Modell. Simul. Mater. Sci. Eng.* **2016**, *24* (8), 85017.

(16) Jin, Z.; Li, X.; Lu, K. Formation of Stable Schwarz Crystals in Polycrystalline Copper at the Grain Size Limit. *Phys. Rev. Lett.* **2021**, *127* (13), 136101.

(17) Li, X. Y.; Jin, Z. H.; Zhou, X.; Lu, K. Constrained minimal-interface structures in polycrystalline copper with extremely fine grains. *Science* **2020**, *370* (6518), 831–836.

(18) Mendelson, M. I. Average Grain Size in Polycrystalline Ceramics. *J American Ceramic Society* **1969**, *52* (8), 443–446.

(19) Borovikov, V.; Mendelev, M. I.; King, A. H. Effects of Ag and Zr solutes on dislocation emission from Σ11(332)[110] symmetric tilt grain boundaries in Cu: Bigger is not always better. *Int. J. Plast.* **2018**, *109*,

79–87.
(20) Borovikov, V.; Mendelev, M. I.; King, A. H. Effects of solutes on dislocation nucleation from grain boundaries. *Int. J. Plast.* **2017**, *90*, 146–155.
(21) Turlo, V.; Rupert, T. J. Grain boundary complexions and the strength of nanocrystalline metals: Dislocation emission and propagation. *Acta Mater.* **2018**, *151*, 100–111.
(22) Garg, P.; Pan, Z.; Turlo, V.; Rupert, T. J. Segregation competition and complexion coexistence within a polycrystalline grain boundary network. *Acta Mater.* **2021**, *218*, 117213.
(23) Hu, Y. C.; Li, F. X.; Li, M. Z.; Bai, H. Y.; Wang, W. H. Five-fold symmetry as indicator of dynamic arrest in metallic glass-forming liquids. *Nat. Commun.* **2015**, *6*, 8310.
(24) Zhu, W.; Li, Z.; Shu, H.; Gao, H.; Wei, X. Amorphous alloys surpass E/10 strength limit at extreme strain rates. *Nat. Commun.* **2024**, *15* (1), 1717.
(25) Wang, N.; Ding, J.; Yan, F.; Asta, M.; Ritchie, R. O.; Li, L. Spatial correlation of elastic heterogeneity tunes the deformation behavior of metallic glasses. *npj Comput Mater* **2018**, *4* (1).
(26) Li, X.; Wei, Y.; Lu, L.; Lu, K.; Gao, H. Dislocation nucleation governed softening and maximum strength in nano-twinned metals. *Nature* **2010**, *464* (7290), 877–880.
(27) Ke, X.; Ye, J.; Pan, Z.; Geng, J.; Besser, M. F.; Qu, D.; Caro, A.; Marian, J.; Ott, R. T.; Wang, Y. M.; Sansoz, F. Ideal maximum strengths and defect-induced softening in nanocrystalline-nanotwinned metals. *Nat. Mater.* **2019**, *18* (11), 1207–1214.
(28) Zhou, H.; Li, X.; Qu, S.; Yang, W.; Gao, H. A jogged dislocation governed strengthening mechanism in nanotwinned metals. *Nano Lett.* **2014**, *14* (9), 5075–5080.
(29) Schiøtz, J.; Jacobsen, K. W. A maximum in the strength of nanocrystalline copper. *Science* **2003**, *301* (5638), 1357–1359.
(30) Stukowski, A. Visualization and analysis of atomistic simulation data with OVITO–the Open Visualization Tool. *Modell. Simul. Mater. Sci. Eng.* **2010**, *18* (1), 15012.
(31) Gupta, A.; Gruber, J.; Rajaram, S. S.; Thompson, G. B.; McDowell, D. L.; Tucker, G. J. On the mechanistic origins of maximum strength in nanocrystalline metals. *npj Comput Mater* **2020**, *6* (1).
(32) Zhao, J. T.; Zhang, J. Y.; Yuan, H. Z.; Wu, K.; Liu, G.; Sun, J. Alloying effects on ductility of nanostructured Cu-X (X = Zr and W) thin films. *Scr. Mater.* **2018**, *152*, 146–149.
(33) Sansoz, F.; Ke, X. Hall–Petch strengthening limit through partially active segregation in nanocrystalline Ag-Cu alloys. *Acta Mater.* **2022**, *225*, 117560.
(34) Peng, H. R.; Jian, Z. Y.; Liu, C. X.; Huang, L. K.; Ren, Y. M.; Liu, F. Uncovering the softening mechanism and exploring the strengthening strategies in extremely fine nanograined metals: A molecular dynamics study. *J. Mater. Sci. Technol.* **2022**, *109*, 186–196.
(35) Panzarino, J. F.; Pan, Z.; Rupert, T. J. Plasticity-induced restructuring of a nanocrystalline grain boundary network. *Acta Mater.* **2016**, *120*, 1–13.
(36) Panzarino, J. F.; Rupert, T. J. Tracking Microstructure of Crystalline Materials: A Post-Processing Algorithm for Atomistic Simulations. *JOM* **2014**, *66* (3), 417–428.
(37) Garbacz, A.; Grabski, M. W. The relationship between texture and CSL boundaries distribution in polycrystalline materials—I. The grain boundary misorientation distribution in random polycrystal. *Acta Metall. Mater.* **1993**, *41* (2), 469–473.
(38) Mason, J. K.; Schuh, C. A. The generalized Mackenzie distribution: Disorientation angle distributions for arbitrary textures. *Acta Mater.* **2009**, *57* (14), 4186–4197.
(39) Barr, C. M.; Foiles, S. M.; Alkayyali, M.; Mahmood, Y.; Price, P. M.; Adams, D. P.; Boyce, B. L.; Abdeljawad, F.; Hattar, K. The role of grain boundary character in solute segregation and thermal stability

of nanocrystalline Pt-Au. *Nanoscale* **2021**, *13* (6), 3552–3563.
(40) Li, L.; Kamachali, R. D.; Li, Z.; Zhang, Z. Grain boundary energy effect on grain boundary segregation in an equiatomic high-entropy alloy. *Phys. Rev. Materials* **2020**, *4* (5).
(41) Peng, H. L.; Li, M. Z.; Wang, W. H. Structural signature of plastic deformation in metallic glasses. *Phys. Rev. Lett.* **2011**, *106* (13), 135503.
(42) Wagih, M.; Schuh, C. A. Spectrum of grain boundary segregation energies in a polycrystal. *Acta Mater.* **2019**, *181*, 228–237.
(43) McLean, D.; Maradudin, A. *Grain boundaries in metals*; American Institute of Physics, 1958.
(44) Jiang, Y.; Li, S.; Liang, N.; Lan, S.; Zhang, Y.; Yin, W.; Sha, G.; Divinski, S.; Wilde, G.; Wang, J. T. Revealing the high strength and high thermal stability of a nano-lamellar Cu-0.1 at.% Zr alloy. *Acta Mater.* **2024**, *276*, 120163.
(45) Zhang, J.; Wang, A.; Zhang, Y.; Ji, J.; Liang, T.; Li, H.; Xie, J. Revealing the influence of solute segregation on the stability and strength of Cu Σ11 [110](113) symmetrical tilt grain boundary via first-principles investigation. *J. Mater. Res. Technol.* **2023**, *26*, 7072–7081.
(46) Wagih, M.; Schuh, C. A. Grain boundary segregation beyond the dilute limit: Separating the two contributions of site spectrality and solute interactions. *Acta Mater.* **2020**, *199*, 63–72.
(47) Pan, Z.; Rupert, T. J. Spatial variation of short-range order in amorphous intergranular complexions. *Comput. Mater. Sci.* **2017**, *131*, 62–68.
(48) Garg, P.; Rupert, T. J. Grain incompatibility determines the local structure of amorphous grain boundary complexions. *Acta Mater.* **2023**, *244*, 118599.
(49) Finney, J. L. Random packings and the structure of simple liquids. I. The geometry of random close packing. *Proc. Phys. Soc. London, Sect. A* **1970**, *319* (1539), 479–493.
(50) Weeks, W. P.; Flores, K. M. Structural building-blocks of disordered Cu-Zr alloys. *Acta Mater.* **2024**, *265*, 119624.
(51) Wei, Y. D.; Peng, P.; Yan, Z. Z.; Kong, L. T.; Tian, Z. A.; Dong, K. J.; Liu, R. S. A comparative study on local atomic configurations characterized by cluster-type-index method and Voronoi polyhedron method. *Comput. Mater. Sci.* **2016**, *123*, 214–223.
(52) Cheng, Y. Q.; Cao, A. J.; Sheng, H. W.; Ma, E. Local order influences initiation of plastic flow in metallic glass: Effects of alloy composition and sample cooling history. *Acta Mater.* **2008**, *56* (18), 5263–5275.
(53) Ding, J.; Ma, E. Computational modeling sheds light on structural evolution in metallic glasses and supercooled liquids. *npj Comput Mater* **2017**, *3* (1).
(54) Zhang, Z.; Ódor, É.; Farkas, D.; Jóni, B.; Ribárik, G.; Tichy, G.; Nandam, S.-H.; Ivanisenko, J.; Preuss, M.; Ungár, T. Dislocations in Grain Boundary Regions: The Origin of Heterogeneous Microstrains in Nanocrystalline Materials. *Metall. Mater. Trans. A* **2020**, *51* (1), 513–530.
(55) Chu, S.; Liu, P.; Zhang, Y.; Wang, X.; Song, S.; Zhu, T.; Zhang, Z.; Han, X.; Sun, B.; Chen, M. In situ atomic-scale observation of dislocation climb and grain boundary evolution in nanostructured metal. *Nat. Commun.* **2022**, *13* (1), 4151.
(56) Farkas, D. Atomistic simulations of metallic microstructures. *Curr. Opin. Solid State Mater. Sci.* **2013**, *17* (6), 284–297.
(57) Wu, S.; Kou, Z.; Lai, Q.; Lan, S.; Katnagallu, S. S.; Hahn, H.; Taheriniya, S.; Wilde, G.; Gleiter, H.; Feng, T. Dislocation exhaustion and ultra-hardening of nanograined metals by phase transformation at grain boundaries. *Nat. Commun.* **2022**, *13* (1), 5468.
(58) Borodin, E. N.; Mayer, A. E.; Gutkin, M. Coupled model for grain rotation, dislocation plasticity and

grain boundary sliding in fine-grained solids. *Int. J. Plast.* **2020**, *134*, 102776.
(59) Wan, L.; Geng, W. T.; Ishii, A.; Du, J.-P.; Mei, Q.; Ishikawa, N.; Kimizuka, H.; Ogata, S. Hydrogen embrittlement controlled by reaction of dislocation with grain boundary in alpha-iron. *Int. J. Plast.* **2019**, *112*, 206–219.
(60) Mendelev, M. I.; Kramer, M. J.; Ott, R. T.; Sordelet, D. J. Molecular dynamics simulation of diffusion in supercooled Cu–Zr alloys. *Philos. Mag.* **2009**, *89* (2), 109–126.
(61) Mattern, N.; Schöps, A.; Kühn, U.; Acker, J.; Khvostikova, O.; Eckert, J. Structural behavior of CuxZr100−x metallic glass (x=35−70). *J. Non-Cryst. Solids* **2008**, *354* (10-11), 1054–1060.
(62) Mendelev, M. I.; Kramer, M. J.; Ott, R. T.; Sordelet, D. J.; Yagodin, D.; Popel, P. Development of suitable interatomic potentials for simulation of liquid and amorphous Cu–Zr alloys. *Philos. Mag.* **2009**, *89* (11), 967–987.
